# Numerical exploration of low lunar, long-lifetime orbits

**DOI:** 10.1007/s10569-026-10334-x

**Published:** 2026-10-04

**Authors:** Natasha Bosanac, Giuliana E. Miceli

**Affiliations:** Colorado Center for Astrodynamics Research, Smead Department of Aerospace Engineering Sciences, University of Colorado Boulder, 3775 Discovery Dr., Boulder, CO 80303 USA

**Keywords:** Low lunar orbits, Frozen orbits, Long-lifetime lunar orbits, Clustering, High-fidelity lunar gravity model

## Abstract

Low lunar orbits that persist over a long duration without impacting the Moon continue to interest the astrodynamics community for trajectory design and space situational awareness. To support exploratory analysis of these low lunar, long-lifetime trajectories in a high-fidelity dynamical model, this paper uses a clustering-based approach. First, low lunar trajectories are numerically generated from coarsely sampled initial conditions for up to 180 days in a 100×100 lunar gravity model along with the point mass gravity of the Earth and Sun. Each trajectory is represented by a subset of its perilunes in a Moon-fixed frame. Distributed trajectory clustering is then used to group trajectories with a similar evolution of eccentricity and argument of perilune, regardless of their initial phasing. From selected global clusters, trajectories with a low level of drift in their evolution of perilune over 180 days and geometrically distinct perilune groundtracks are isolated as long-lifetime orbits. Sets of geometrically similar long-lifetime orbits are presented, analyzed, and then coarsely compared to analytically derived frozen orbit predictions from the existing literature.

**Supplementary Information:**

The online version contains supplementary material available at https://doi.org/10.1007/s10569-026-10334-x.

## Introduction

Low lunar orbits that maintain a limited evolution of their size, shape, and/or orientation over a long duration have been of significant interest to the astrodynamics community over the last few decades. A subset of these trajectories are frozen orbits which maintain near constant mean values of the eccentricity, argument of perilune, and inclination in a Moon-fixed frame (Herder et al. 1976; Folta et al. 1998), consistent with a nearly quasi-periodic evolution of perilune (Folta and Quinn 2006). This special class of lunar orbits has been studied by a variety of researchers, building upon prior analyses for Earth-orbiting spacecraft (Coffey et al. 1994; Broucke 2003). Frozen orbits have also been studied around other celestial bodies, e.g., Mercury (Lara et al. 2010) and Europa (Paskowitz and Scheeres 2006).

Low lunar frozen orbits may be useful when designing missions for extended scientific observations of the lunar surface or placement of critical infrastructure while maintaining low station-keeping maneuver requirements. For instance, Lunar Prospector (Folta et al. 1998) and the Lunar Reconnaissance Orbiter (LRO) (Beckman 2006) were placed into polar, frozen orbits. Through careful selection of the reference orbits, LRO used only 33.9 kg of hydrazine propellant over 12 years for station-keeping maneuvers, phasing maneuvers, and momentum unloads combined (Mesarch 2023). Lunar frozen orbits may also be useful for locating debris (Boone and Bettinger 2023) or older spacecraft (Meador 2021) that may orbit the Moon over long durations.

A common approach to identifying lunar frozen orbits involves analytically constructing an averaged dynamical system that removes short- and/or medium-period effects and then identifying equilibrium solutions. Early analyses, such as those by Park and Junkins (1994), Ely (2005), and Folta and Quinn (2006), used the Lagrange planetary equations in dynamical models with low degree and order spherical harmonic lunar gravity fields and/or third-body perturbations from the Earth. Alternatively, Lara (2011) as well as Nie and Gurfil (2018) formulated a Hamiltonian system in terms of Delauney variables. Lara (2011) used spherical harmonic lunar gravity models up to a degree and order of 100×100 along with point mass gravity of the Earth in a circular orbit aligned with the equatorial plane of the Moon; this analysis also supported exploring the connection between dynamical model fidelity and the characteristics of lunar frozen orbits. Through these analytical approaches, most of these papers have presented curves or surfaces relating predicted mean values of eccentricity, inclination in a Moon-fixed frame, argument of perilune in a Moon-fixed frame, and semi-major axis across families of lunar frozen orbits. These papers have noted special cases where lunar frozen orbits exist, including (1) a mean argument of perilune equal to $\pm 90^{\circ }$ (Park and Junkins 1994; Lara 2011), (2) a mean eccentricity of zero (Nie and Gurfil 2018), or (3) a mean argument of perilune equal to $0^{\circ }$ or $180^{\circ }$ (Nie and Gurfil 2018). The associated initial conditions and nearby trajectories are then usually simulated in a higher-fidelity model for further analysis of selected point solutions.

More recent approaches to studying lunar frozen orbits focus on numerically generating periodic solutions to approximate dynamical models via integration and differential correction. For instance, Elipe and Lara (2003) generated families of frozen orbits by identifying periodic solutions in the radial and vertical components of cylindrical coordinates for a spacecraft governed by a lunar gravity field with the first seven zonal terms. Russell and Lara (2007) later identified repeat groundtrack orbits by numerically generating families of periodic orbits in the Earth-Moon circular restricted three-body problem with a 50×50 lunar gravity model.

An alternative numerical approach involves examining lunar frozen orbits via trajectories that are numerically generated in a high-fidelity ephemeris model from various initial orbital elements. For instance, Legnaro and Efthymiopoulos (2024) graphically represented the fast Lyapunov indicator and duration of trajectories before lunar impact from a variety of initial eccentricities, inclinations, and semi-major axes. Alternatively, Plice et al. (2017) generated near-circular, near-equatorial trajectories in a 100×100 gravity model with third-body effects. Then, they identified frozen orbits by manually examining variations in the perilune altitude and longitude via a contour map. Extending this approach to a wider region of the phase space offers the benefit of identifying lunar frozen orbits in high-fidelity models with fewer assumptions than an analytical approach. However, a significant challenge emerges: extracting frozen orbits with an array of distinct geometries and additional meaningful insights from a large dataset. This challenge in analyzing large datasets without overburdening an analyst or relying on a priori information is shared across the broader scientific community and has inspired the use of data mining techniques.

Clustering is a data mining technique that automatically groups similar data into a cluster and separates dissimilar data (Han et al. 2006). These groupings supply a summary of the dataset that (1) shifts the workload for exploratory data analysis from low-level sorting tasks to analysis of a smaller number of groups that sufficiently represent the data, or (2) supports automating subsequent decision-making. Clustering has proven valuable to a variety of disciplines: in medicine, clustering has been used to detect clinically meaningful shape clusters in medical image data (Bruse et al. 2017) whereas in astronomy, clustering has been used to identify distinct types of galaxies (Tohill et al. 2023). In astrodynamics, clustering has been used to detect bounded motions near distant retrograde orbits (Nakhjiri and Villac 2015), group periodic orbits that are independently computed (Villac et al. 2016), extract motion primitive sets that summarize geometrically similar segments (Smith and Bosanac 2022), and summarize a diverse array of nonlinear trajectories in the circular restricted three-body problem by their geometry (Bosanac 2026).

This paper presents a proof of concept for using clustering to extract groups of geometrically similar low lunar trajectories that are numerically generated in a high-fidelity lunar gravity model for up to 180 days. This clustering result is used to guide manual selection of long-lifetime orbits, defined as trajectories with a limited perilune drift over 180 days. Some, but not necessarily all long-lifetime orbits, may lie close to frozen orbits and may serve as a useful starting point for further analysis or differential corrections. First, over one million initial perilunes are defined using coarsely sampled orbital elements at a specific initial epoch with initial semi-major axes no larger than 2,038 km. Trajectories are then generated in an ephemeris model for up to 180 days from these initial conditions with a 100×100 lunar gravity model as well as the point mass gravity of the Earth and Sun. From these trajectories, 638,181 possess durations of at least three lunar rotational periods and analyzed further. Distributed trajectory clustering is then used to group these trajectories when their perilune sequences exhibit a geometrically similar evolution in a Moon-fixed frame, regardless of their initial phasing. Selected clusters are then individually analyzed to extract sets of geometrically distinct long-lifetime orbits that exist at discrete values of initial inclination and semi-major axis. In some cases, varying the initial longitude of the ascending node causes the perilunes of long-lifetime orbits with a similarly shaped evolution of eccentricity and argument of perilune to trace out geometrically distinct groundtracks.

This clustering approach to large-scale numerical exploration of low lunar trajectories in a high-fidelity ephemeris model offers multiple contributions. First, this paper demonstrates that a clustering problem can be formulated to extract meaningful groups of trajectories with a geometrically similar evolution of eccentricity and argument of perilune. With limited bias from a priori insight into the solution space, 638,181 trajectories are summarized by 885 global clusters, reducing the amount of information to analyze. Following manual analysis of the global cluster summary, geometrically distinct sets of long-lifetime orbits are presented via their evolution of eccentricity and argument of perilune as well as the associated perilune groundtracks. These results may support selecting mission orbits that naturally exhibit a low drift over multiple months with a desired geometry. Some long-lifetime orbits even exhibit a low-order resonance or possess distinct groundtrack geometries at the same initial semi-major axis and inclination. Although generated over a shorter duration, a subset of the long-lifetime orbits demonstrate consistency with previous, analytically derived frozen orbit conditions. In this case, these trajectories supply an initial guess for future differential corrections to produce lower-drift solutions over longer durations.

## Dynamical model

Two Moon-centered reference frames are used to describe the state of a spacecraft. The inertial frame uses the axes $\hat{X},\hat{Y},\hat{Z}$ of the International Celestial Reference Frame (ICRF) (International Earth Rotation and Reference Systems Service 2010). In addition, a Moon-fixed frame is defined using the Moon’s principal axes; note that the first two axes lie close to the lunar equatorial plane (Lunar Reconnaissance Orbiter Project and Lunar Geodesy and Cartography Working Group 2008). The transformation between the inertial and principal axes is calculated using the Spacecraft, Planet, Instrument, C-matrix, Events (SPICE) toolkit and the moon_080317 kernel file from NASA’s Navigation and Ancilliary Information Facility, which is compatible with the DE421 lunar and planetary ephemerides (Acton 1996; Acton et al. 2018).

Trajectories are generated in a high-fidelity lunar gravity model augmented by the point mass gravity of the Earth and Sun. In the Moon-centered inertial frame, the state vector for the spacecraft relative to the Moon is defined as $[X,Y,Z,\dot{X},\dot{Y},\dot{Z}]^T=[\boldsymbol{R}_{L,sc},\boldsymbol{V}_{L,sc}]^{T}$, where $\boldsymbol{R}_{L,sc}$ and $\boldsymbol{V}_{L,sc}$ are the position and velocity vectors of the spacecraft relative to the Moon. The dot notation indicates a time derivative with respect to an observer fixed in the inertial frame. The spacecraft is also assumed to possess a negligibly small mass compared to the celestial bodies. With this assumption, the equations of motion governing the spacecraft (Vallado 2013) are written as

1 $$\begin{aligned} \ddot{\boldsymbol{R}}_{L,sc} = -GM_{L}\left( \frac{\boldsymbol{R}_{L,sc}}{R^{3}_{L,sc}}\right) + G \sum _{i=E,S} M_{i}\left( \frac{\boldsymbol{R}_{sc,i}}{R^{3}_{sc,i}} - \frac{\boldsymbol{R}_{L,i}}{R^{3}_{L,i}} \right) + \boldsymbol{a}_{L} \end{aligned}$$

where the subscripts *L*, *E*, *S*, and *sc* denote the Moon, Earth, Sun, and spacecraft, respectively; $M_i$ is the mass of body *i*; *G* is the universal gravitational constant; $\boldsymbol{R}_{i,j}$ is the position vector from body *i* to body *j*; and $\boldsymbol{a}_{L}$ is the acceleration due to higher-order lunar gravity terms. The DE421 lunar and planetary ephemerides are used to locate each celestial body at each epoch (Folkner et al. 2009). Additional perturbing accelerations are not modeled in this paper, but may be useful for future work.

The lunar gravity field is modeled by a 100×100 degree and order spherical harmonics model. For a position vector located above the Moon’s surface, the potential function $U_L$ for the deviation of the gravity field from a point mass is expressed in the Moon’s principal axes as

2 $$\begin{aligned} U_{L} = \frac{GM_{L}}{R_{L,sc}} \left[ \sum _{l=2}^{100} \sum _{m=0}^{l}\left( \frac{R_{L}}{R_{L,sc}} \right) ^{l} P_{l,m}\left( \sin (\phi ) \right) \left( C_{l,m}\cos (m\lambda ) + S_{l,m}\sin (m\lambda ) \right) \right] \end{aligned}$$

where $R_{L}$ is the reference radius of the Moon, $P_{l,m}$ is the associated Legendre polynomial for degree *l* and order *m*, ϕ and λ are the selenocentric latitude and longitude, and $C_{l,m}$ and $S_{l,m}$ are the coefficients of the spherical harmonic expansion (Vallado 2013). In this work, the coefficients of expansion and model parameters are accessed using the 900×900 GRGM900C gravity model which is compatible with the DE421 ephemerides (Lemoine et al. 2014). Then, a frame transformation is applied to the vector derivative of $U_{L}$ to calculate the acceleration $\boldsymbol{a}_{L}$ in the Moon-centered inertial frame for use in Eq. (1). Although higher degree and order gravity coefficients are available, they are not included due to (1) their significant influence on the computational time and (2) the Lunar Prospector mission’s successful use of a quasi-frozen orbit that was identified in a 100×100 gravity model (Konopliv et al. 2001).

## Trajectory clustering

Trajectory clustering groups a set of moving objects if they follow similar paths (Zheng and Zhou 2011). One approach groups trajectories, each represented by a time series approximation, if they consistently remain within each others’ local neighborhoods at the sampled times (Zheng and Zhou 2011). First, the sampled variables at a similar time along all trajectories are described in a selected feature vector space, e.g., a set of Cartesian coordinates. Density-based clustering is then applied to these feature vectors to identify groups of samples that exist in sufficiently dense local neighborhoods (Zheng and Zhou 2011; Jeung et al. 2008). This process is repeated to produce a sequence of clustering results, each localized to a specific instant (Zheng and Zhou 2011; Jeung et al. 2008). Combining all clustering results, a group of trajectories is formed if they (1) appear in the same cluster over multiple time instants, and (2) exceed a minimum membership size. This group has been labeled as a swarm if the trajectories are clustered together for at least a minimum number of nonconsecutive time instants (Zheng and Zhou 2011), allowing the trajectories to briefly deviate but generally follow a similar path over long durations.

A well-known clustering algorithm is the Density-Based Spatial Clustering of Applications with Noise (DBSCAN). Developed by Ester et al. (1996), DBSCAN discovers groups of data with a density that exceeds a specified threshold. The members of a dataset, each described in a selected feature vector space, are categorized as one of three types of points (Ester et al. 1996):

- Core points possess at least $m_{pts}$ neighbors within a distance of ϵ. Thus, core points possess local neighborhoods with a sufficient density.
- Border points are located within a distance of ϵ from a core point but possess fewer than $m_{pts}$ neighbors within a distance of ϵ.
- Noise points do not lie within a distance of ϵ from any core points.

Using these definitions, each cluster is constructed by first identifying core points that lie within a distance of ϵ from another core point (Ester et al. 1996). Then, border points are added to the clusters of their associated core point. Accordingly, each of the members of a dataset are either assigned to a cluster or designated as noise (Ester et al. 1996). There are two governing parameters, $m_{pts}$ and ϵ, and DBSCAN is accessed via the *dbscan* function in MATLAB (The MathWorks Inc 2023).

## Technical approach

This section presents a clustering-based approach to summarizing a large set of low lunar trajectories, generated numerically in a high-fidelity dynamical model. This approach consists of the following steps:

1. Numerically generate a set of trajectories.
2. Describe each trajectory by the characteristics of its perilune sequence.
3. Cluster individual partitions of the dataset to produce groups of spatiotemporally similar trajectories.
4. Merge clusters with spatiotemporally similar trajectories across distinct partitions and/or geometrically similar trajectories with distinct phasing.
5. Extract long-lifetime orbits with a low drift from selected clusters.

A summary of the parameters governing the process, described in detail throughout this section, is provided in Online Resource 2.

### Step 1: generating a dataset of low lunar trajectories

To define a dataset of low lunar trajectories, initial conditions are formed using Keplerian orbital elements. To limit the size of the dataset and computational time, each initial condition is defined as a perilune at a fixed initial epoch, $T_0$; however, varying $T_0$ is an interesting avenue for future work. The initial orbital elements are then specified via ranges and step sizes of the semi-major axis *a*, eccentricity *e*, inclination *i*, longitude of the ascending node Ω, and argument of perilune ω. A subscript of ‘0’ throughout the paper indicates an initial value. Each unique combination of these orbital elements is used to generate a candidate six-dimensional, inertial state vector. Only state vectors that lie above the Moon’s surface, modeled as a sphere with a radius of 1,738 km (Folkner et al. 2009), are retained for further use.

Each retained inertial state vector is propagated in the ephemeris model described in Sect. 2. The propagator uses the Runge–Kutta Prince-Dormand (8, 9) method in the GNU Scientific Library, accessed via C++ (Galassi et al. 2009). The position, velocity, and time are specified in units of km, km/s, and seconds, respectively, with absolute and relative integration tolerances of $10^{-11}$. In this paper, the maximum duration for each trajectory is selected as 180 days to balance (1) generating the trajectories for long enough to observe any drift, (2) identifying patterns in the perilune evolution over a similar time scale to the lunar rotational period, and (3) reducing the computational time and storage. However, trajectories terminate early upon impact with a spherical approximation of the Moon with a radius of 1,738 km (Folkner et al. 2009). In this paper, each trajectory requires up to several minutes to generate on a Linux computer with a processing speed of 2.85 GHz per core.

To limit data storage and capture longer period variations, each trajectory is represented by a sequence of perilunes (Folta and Quinn 2006; Russell and Lara 2007; Lara 2011; Nie and Gurfil 2018). These perilune events are identified during propagation using a foundational bisection method (Press et al. 2007) and labeled as a perilune sequence. If a perilune sequence spans a duration of at least three lunar rotational periods, i.e., 81.9 days, it is added to the trajectory dataset. This threshold ensures the applicability of later steps of the clustering process.

To reduce the computational burden of clustering a large dataset, the dataset is distributed across multiple, smaller partitions; such an approach is commonly used in distributed clustering (Aggarwal and Reddy 2018). First, the trajectories are sorted by their initial orbital elements in the following order: *i*, *a*, ω, Ω, *e*. Then, the trajectories are equally distributed across ρ partitions with no more than 10,000 members.

### Step 2: describe each perilune sequence

#### Two-dimensional coordinate representations

To support identifying groups of low lunar trajectories with a geometrically similar evolution, each perilune is described using two different two-dimensional representations. The following two sets of variables are used:

1. The variables $p = {e}\cos ({\omega })$ and $q={e}\sin ({\omega })$ capture both the instantaneous shape of an orbit and the orientation of the perilune within the orbit; note that ω is calculated in the lunar principal axes. These two coordinates have commonly been used to visualize the evolution of perilune when examining lunar frozen orbits and repeat groundtrack orbits (Herder et al. 1976; Lara 2011; Nie and Gurfil 2018; Russell and Lara 2007). Furthermore, the coordinates *p* and *q* possess similar value ranges, which is useful during later clustering steps.
2. The selenocentric longitude λ and latitude ϕ in the lunar principal axes are used to visualize the groundtrack of the perilune sequence.

Throughout this paper, the groundtrack of the perilune sequence is labeled as a perilune groundtrack and is plotted using one of the following two projections:

1. The Lambert azimuthal equal-area projection is used when the perilunes do not revolve above a polar region. A latitude of zero appears as a horizontal line and a selected central meridian appears as a vertical line (Snyder and Voxland 1989). The meridians intersect at $\phi =\pm 90^{\circ }$ (Snyder and Voxland 1989).
2. The polar stereographic projection is used when the perilune groundtrack maintains a consistently high latitude (McClernan et al. 2025). Centered on a latitude of $\phi =\pm 90^{\circ }$, meridians appear as radial lines whereas concentric circles capture lines of constant latitude (Snyder and Voxland 1989).

#### Sampling the perilune sequence

Each perilune sequence is sampled to (1) construct a time series representation and (2) reduce computational time and storage. Consider a trajectory that passes through $N_{orig}$ perilunes with the final perilune occurring T∈[81.96,180] days after the initial epoch. The $N_{samp}=\lfloor T/ \Delta t\rfloor$ perilunes that occur closest to every Δt=0.5 day increment are sampled, with $163 \le N_{samp} \le 360$. These samples are labeled as a sampled perilune sequence.

#### Feature vector description

A feature vector is defined to describe the shape of a perilune sequence in the *pq*-plane at the *i*th sample. This *i*th sampled perilune corresponds to the *j*th perilune along the original sequence and occurs at an epoch $t_j$; this index transformation is denoted as *j*(*i*). Forward finite difference approximations of the time derivative of the *pq*-coordinates along the original perilune sequence are calculated at this perilune as

3 $$\begin{aligned} \dot{\tilde{p}}_{j(i)} = \frac{p_{j+1}-p_{j}}{t_{j+1}-t_{j}} \qquad \dot{\tilde{q}}_{j(i)} = \frac{q_{j+1}-q_{j}}{t_{j+1}-t_{j}} \end{aligned}$$

for an integer $j \in [1, N_{orig}-1]$. Then, the feature vector $\boldsymbol{f}_{\dot{p}\dot{q},i}$ is defined as the tangent vector at the *i*th perilune along the sampled perilune sequence in the *pq*-plane as

4 $$\begin{aligned} \boldsymbol{f}_{\dot{p}\dot{q},i} = \left[ \frac{\dot{\tilde{p}}_{j(i)}}{\sqrt{\left( \dot{\tilde{p}}_{j(i)}^2+\dot{\tilde{q}}_{j(i)}^2\right) }} \qquad \frac{\dot{\tilde{q}}_{j(i)}}{\sqrt{\left( \dot{\tilde{p}}_{j(i)}^2+\dot{\tilde{q}}_{j(i)}^2\right) }} \right] = \left[ \dot{\tilde{P}}_{j(i)} \qquad \dot{\tilde{Q}}_{j(i)} \right] \end{aligned}$$

This feature vector is a two-dimensional unit vector.

#### Approximating drift and period of oscillation

As observed in the existing literature, the perilune sequence tends to exhibit a prominent oscillation that is on a similar timescale to the lunar rotational period (Folta and Quinn 2006; Russell and Lara 2007). The period of this oscillation, $T_{osc}$, is estimated using the shape of the perilune sequence in the *pq*-plane. First, the $N_{r}$ perilunes that occur within the first 14 days are extracted from the original perilune sequence and labeled as a reference subsequence. Additional subsequences of $N_{r}$ perilunes are sampled to begin at each perilune with an epoch T>14 days. The Euclidean distance $d_{shape,j}$ is calculated between all $N_{r}$ perilunes along the reference subsequence and the candidate subsequence that begins from the *j*th perilune in $\boldsymbol{f}_{\dot{p}\dot{q}}$ as

5 $$\begin{aligned} d_{shape,j} = \sum _{i=1}^{N_{r}} \sqrt{\left( \dot{\tilde{P}}_{ref,i}- \dot{\tilde{P}}_{cand,j+i-1} \right) ^2 + \left( \dot{\tilde{Q}}_{ref,i}- \dot{\tilde{Q}}_{cand,j+i-1} \right) ^2} \end{aligned}$$

where the subscripts ‘ref’ and ‘cand’ identify the reference and candidate subsequences, respectively. This distance compares the shape of the two subsequences in the *pq*-plane and oscillates with a period $T_{osc}$ as *j* is increased. The elapsed time between the first two dominant minima, separated by at least 21 days, supplies an estimate for $T_{osc}$.

The drift $d_{pq}$ of a perilune sequence over 180 days is estimated as the distance between the first and final full revolutions in the *pq*-plane. First, the $N_{f}$ perilunes within $T_{osc}$ of the initial epoch of the original perilune sequence represent the first full revolution. Then, the $N_{f}$ perilunes at epochs $T\in [(\alpha -1)T_{osc},\alpha T_{osc}]$, where $\alpha =\lfloor {T/T_{osc}\rfloor }$, represent the final full revolution. The cumulative distance $d_{pq}$ is calculated between the $N_{f}$ perilunes along each revolution in an isochronous manner as

6 $$\begin{aligned} d_{pq} = \sum _{i=1}^{N_{f}} \sqrt{\left( p_{1,i}- p_{f,i} \right) ^2 + \left( q_{1,i}- q_{f,i} \right) ^2} \end{aligned}$$

where the subscripts ‘1’ and ‘f’ identify the first and final revolutions.

The exception to the drift calculation is a perilune sequence that exhibits a low-order resonance. These special cases are identified when the drift between subsequent revolutions in the *pq*-plane oscillates with a period that is less than 180 days. In this case, $d_{pq}$ is calculated as the difference between the *pq*-coordinates of the perilunes along the first revolution and the nearest subsequent revolution.

### Step 3: clustering individual partitions of trajectories

Trajectories are clustered by the shape and initial phasing of their sampled perilune sequences in the *pq*-plane. In this paper, a group of spatiotemporally similar perilune sequences is defined using the concept of a swarm from trajectory clustering: the *i*th sampled perilunes along the grouped trajectories must lie within sufficiently dense neighborhoods in $\boldsymbol{f}_{\dot{p}\dot{q}}$ for at least $N_{thresh}$ nonconsecutive samples. To identify these groups, a spacecraft trajectory clustering approach recently presented by Bosanac (2026) is used with minor modifications.

To describe the clustering process, consider the *j*th partition that is composed of *M* perilune sequences, each described by between $N_{min}$ and $N_{max}$ sampled perilunes. The feature vector $\boldsymbol{f}_{\dot{p}\dot{q},i}$, is evaluated at the *i*th perilune along each of the *M* sequences. Then, DBSCAN is used to cluster these *M* feature vectors, using the Euclidean distance to assess similarity. If the *i*th sampled perilunes along two sequences exist and satisfy the following two conditions, they are identified as neighbors: (1) they are grouped together by DBSCAN for the selected values of $m_{pts}$ and ϵ, and (2) they exist within a distance of ϵ. This process is repeated for all $N_{max}$ perilunes. If two sequences possess at least $N_{thresh}$ perilunes that are connected by a sequence of neighbors, they are grouped together. Groups with at least $m_{pts}+1$ members form a cluster.

The clustering process produces a set of $K^j_\ell$ local clusters $\mathcal {C}^{j}_{\ell } = \{\mathcal {C}^{j}_{\ell ,1},\mathcal {C}^{j}_{\ell ,2},..., \mathcal {C}^{j}_{\ell ,K^j_\ell } \}$ of spatiotemporally similar perilune sequences and noise $\mathcal {U}^{j}_\ell$ that summarize the *j*th partition. To concisely represent each local cluster, a cluster representative is extracted as the perilune sequence with the lowest drift in the *pq*-plane, possessing the minimum value of $d_{pq}$ as defined in Eq. (6). This process is repeated across all partitions.

When clustering each partition, the parameters governing DBSCAN must be specified. First, the neighborhood size is selected as $m_{pts}=4$ to prioritize discovering local variations across a sparse dataset. Both ϵ and $N_{thresh}$ are then heuristically selected to balance two competing objectives: (1) remaining small (or large) enough to ensure that all perilune sequences in the $m_{pts}$-neighborhood of a core point have a sufficiently similar tangent vector, and (2) being large (or small) enough to identify similar shapes across a sparse dataset and, therefore, avoid designating an excessive number of points as noise. In this paper, $\epsilon =2\sin (10^{\circ })$, to include unit vectors separated by an angle of up to $20^{\circ }$ (Bosanac 2026). Finally, $N_{thresh}=N_{max}-27$ allows two perilune sequences to be grouped if they (1) possess durations that differ by 13.5 days, or (2) exhibit only brief shape differences during each revolution in the *pq*-plane.

### Step 4: aggregating clusters across partitions

Local clusters are aggregated to produce a set of global clusters that summarize the geometries exhibited by the generated set of low lunar trajectories. Aggregation consists of two steps, merging clusters of perilune sequences that exhibit:

1. A similar geometry and phasing but exist within distinct partitions
2. A similar geometry but distinct initial phasing

This procedure is modeled after an approach previously developed by Bosanac (2026).

The first cluster aggregation step merges local clusters across partitions if their perilune sequences possess a similar geometry and phasing. Consider a local cluster representative described by *N* sampled perilunes. A 2*N*-dimensional feature vector is calculated along the representative as $\boldsymbol{f}_{shape}=[\boldsymbol{f}_{\dot{p}\dot{q},1},\boldsymbol{f}_{\dot{p}\dot{q},2},..., \boldsymbol{f}_{\dot{p}\dot{q},N}]$, summarizing the shape of the path in the *pq*-plane. This shape-based feature vector is calculated for the representatives of all local clusters across all partitions. Then, for each local cluster, $\boldsymbol{f}_{shape}$ is compared with the feature vectors of all the other representatives that 1) exist in other partitions, and 2) possess durations that differ by no more than 13.5 days, consistent with the value of $N_{thresh}$. The Euclidean distance is used to assess the difference between each pair of feature vectors, with zeros added to the end of any feature vectors that are shorter than the other. Then, the $k_1$ nearest neighbors in $\boldsymbol{f}_{shape}$ coarsely identify $k_1$ candidate local clusters for merging with the current cluster of interest. These candidate pairs are identified for every local cluster across all partitions, producing up to $k_1K_{\ell }$ pairs where $K_{\ell }=\sum _{j=1}^{\rho }K_{\ell }^j$. Then, perilune sequences from each candidate pair of local clusters are input to the clustering procedure outlined in Step 3. If the two local clusters are assigned to the same group, they are merged.

The second cluster aggregation step identifies local clusters with a similar path in the *pq*-plane with distinct initial phasing. First, the representative perilune sequence of cluster *i* is clipped to remove the first $n_i$ sampled perilunes. Every local cluster with a representative that possesses a duration within at least one lunar rotational period of the representative trajectory of cluster *i* is labeled as a candidate neighbor. Then, the representative trajectory of candidate neighbor *j*, where i≠j, is clipped to remove the last $n_j$ sampled perilunes. The integer values of $n_i$ and $n_j$ are each varied in the range [1, 54], consistent with clipping perilunes for up to one lunar rotational period. For each combination of $n_i$ and $n_j$, $\boldsymbol{f}_{shape}$ is calculated for each clipped perilune sequence of the representatives of clusters *i* and *j*. The Euclidean distance between these feature vectors describing the clipped representatives of clusters *i* and *j* is calculated. This process is repeated to reverse the clipping direction, removing perilunes from the end of cluster *i* and start of cluster *j*. This process is then repeated for all candidate neighbors to cluster *i*, regardless of their partition. The $k_2$ lowest distances when clipping in each direction produce up to $2k_2$ combinations of local clusters and values of $n_i$ and $n_j$ that supply candidates for merging with cluster *i*. This process is repeated for all $K_{\ell }$ local clusters across all partitions to produce up to $2k_2K_{\ell }$ candidate pairs.

Each candidate pair of local clusters for merging across distinct initial phasing is evaluated. The perilune sequences in each local cluster are clipped based on the clipping direction and values of $n_i$ and $n_j$ identified earlier. These clipped trajectories are then clustered using the procedure outlined in Step 3. Because $n_i$ and $n_j$ are coarsely identified, this process is repeated by varying $n_i$ and $n_j$ to within one sampled perilune of the estimated values. If any trajectories from distinct local clusters are grouped together, the two local clusters are merged.

To identify global clusters from the pairwise merging decisions, a graph structure is employed. The $K_{\ell }$ nodes of the graph represent the $K_{\ell }$ local clusters across all partitions. Then, undirected and unweighted edges are added between local clusters that are merged at either aggregation step. Each connected component of this graph defines one of $K_{g}$ global clusters $\mathcal {G} = \{\mathcal {G}_{1},\mathcal {G}_{2},..., \mathcal {G}_{K_g} \}$, each containing perilune sequences with a similar path through the *pq*-plane, regardless of the initial phasing.

### Step 5: extract long-lifetime orbits from global clusters

Visual inspection is used to manually identify global clusters that may contain long-lifetime orbits. Recall that long-lifetime orbits are defined in this paper as trajectories with bounded or limited drift in the evolution of perilune over 180 days; some, but not necessarily all, of these trajectories may lie near frozen orbits. In this paper, the perilune sequence that produces the minimum value of $d_{pq}$ in each global cluster is plotted in the *pq*-plane and visually inspected: if the drift is sufficiently small over 180 days, its global cluster is isolated for further analysis. Due to the sparse nature of the data and variety of solution geometries, the determination of a sufficiently small value of $d_{pq}$ is made subjectively.

One or more subsets of perilune sequences that possess a low drift in the *pq*-plane are extracted from each selected global cluster to (1) approximate long-lifetime orbits, (2) capture their evolution over the semi-major axis, and (3) differentiate between distinct groundtrack geometries. First, the perilune sequences comprising the selected global cluster are subdivided based on $a_0$ and $\Omega _0$. For each combination of $a_0$ and $\Omega _0$, the perilune sequence with the minimum value of $d_{pq}$ is identified and plotted in both the *pq*-plane and its groundtrack. For each value of $a_0$, these plots are visually inspected to determine whether multiple perilune sequences possess distinct values of $\Omega _0$ and trace out paths that either (1) produce groundtracks with a distinct geometry, or (2) exist within distinct regions of the *pq*-plane. The perilune sequences that meet either of these criteria are manually extracted at each value of $a_0$. This process produces one or more sets of representative members with a geometrically similar perilune groundtrack across a range of initial semi-major axes. Automatically and robustly extracting these perilune sequences is an area of ongoing work.

**Fig. 1:**
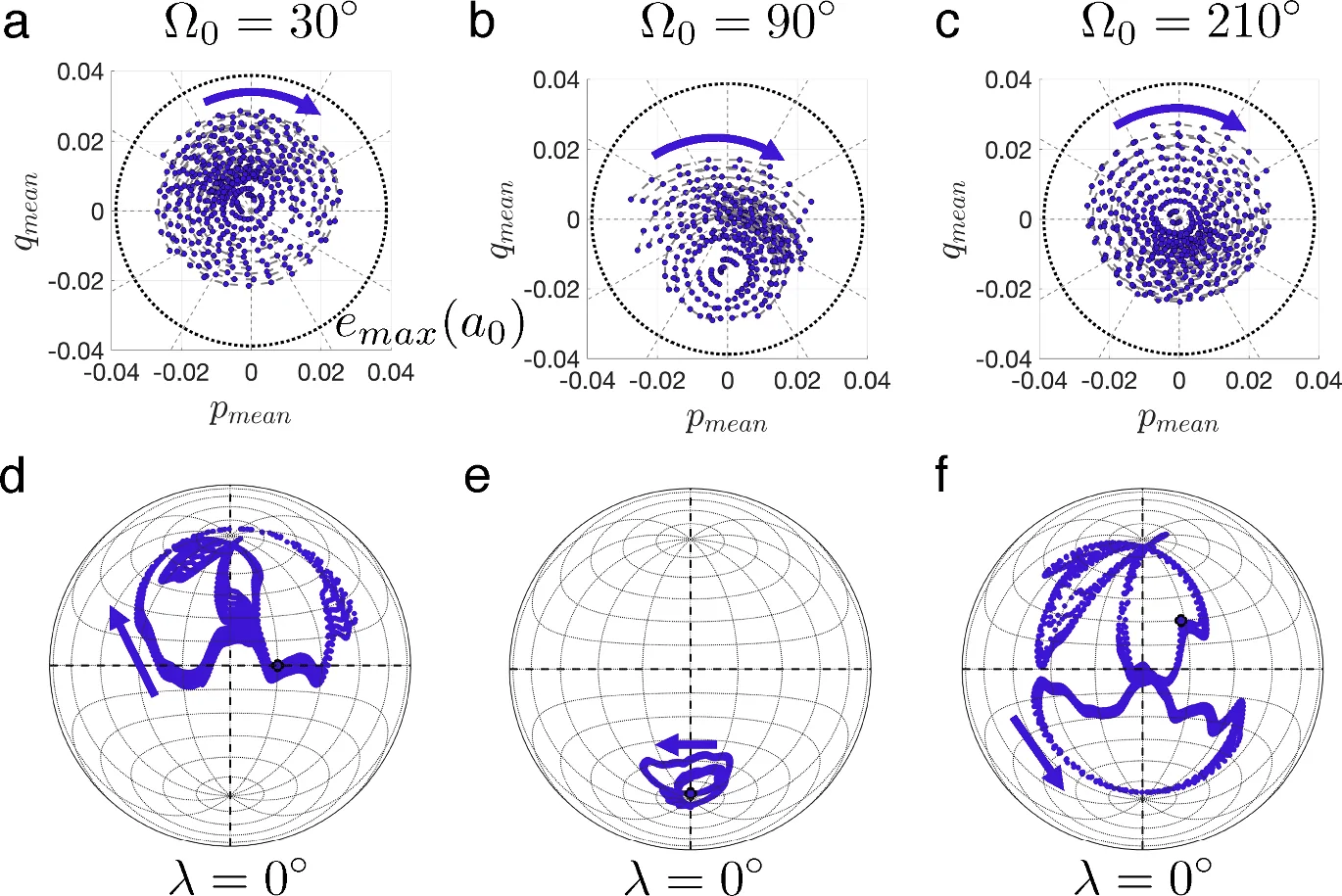
For a set of perilune sequences with a similar shape in the *pq*-plane at $a_0=1,808$ km, $i=85^{\circ }$ at three values of $\Omega _0$: **a**–**c** evolution of $p_{mean}$ and $q_{mean}$, and **d**–**f** perilune groundtracks of selected long-lifetime orbits

Varying the initial longitude of the ascending node can, in some cases, produce substantial variations in the groundtrack geometry at a single initial semi-major axis. To demonstrate this observation, the drift is visualized across perilune sequences belonging to the same cluster. Along each perilune sequence, the average value of *p* and *q* is calculated along each revolution in the *pq*-plane, formed by perilunes with epochs between subsequent integer multiples of $T_{osc}$. These quantities are denoted as $p_{mean}$ and $q_{mean}$ and plotted in the *pq*-plane.

The $p_{mean}$ and $q_{mean}$ plots are similar to the eccentricity vector diagrams examined by Lara (2011) and Yarndley et al. (2020). In the autonomous and averaged dynamical models typically used for analytical predictions, a frozen orbit would appear as a fixed point in a diagram of the mean eccentricity and argument of periapsis or, similarly, $p_{mean}$ and $q_{mean}$, at a single mean inclination and mean semi-major axis. Stable frozen orbits are surrounded by closed curves in $p_{mean}$ and $q_{mean}$ associated with quasi-periodic paths in the *pq*-plane and unstable solutions lie at the asymptotes of hyperbolic curves. Because an ephemeris model is not autonomous or periodic, these characteristics are not exactly observed for the trajectories in this paper. However, similar variations are approximately observed in some plots of $p_{mean}$ and $q_{mean}$.

Consider a cluster of perilune sequences with a similar evolution in the *pq*-plane at $a_0=1,808$ km and $i_0=85^{\circ }$. At each of three selected values of $\Omega _0$, the evolution of $p_{mean}$ and $q_{mean}$ along selected members of this set is represented in Fig. 1a–c with circle markers. Although they are discrete values, gray dashed lines connect $p_{mean}$ and $q_{mean}$ along successive revolutions to convey evolution along a single trajectory. Because early termination via lunar impact typically occurs as the eccentricity becomes large (Folta and Quinn 2006), the dashed circle indicates the maximum possible eccentricity at the initial semi-major axis, denoted as $e_{max}(a_0)$. In each subfigure, long-lifetime orbits produce sequences with little variation in $p_{mean}$ and $q_{mean}$. In these three examples, additional perilune sequences librate around them, suggesting that stable frozen orbits might exist in their vicinity. However, additional trajectories generated from more finely sampled initial orbital elements and/or differential corrections would be needed to confirm this assessment.

**Table 1:** Ranges of orbital elements and selected epoch used to define candidate initial conditions, with all angles defined in the lunar principal axes

Quantity	Range of values	Step size
$T_0$	January 1, 2025 00:00.000 UTC	–
*a*	[1748, 1838] km	10 km
	[1863, 1938] km	25 km
	[1988, 2038] km	50 km
*e*	$[10^{-4}, 0.1]$	0.002−0.005, depending on *a*, when $e > 10^{-4}$
*i*	$[0.001^{\circ }, 5^{\circ },..., 175^{\circ }, 179.999^{\circ }]$	$5^{\circ }$ when $5^{\circ } {\le } i {\le } 175^{\circ }$
Ω	$[0^{\circ }, 360^{\circ })$	$30^{\circ }$
ω	$[0^{\circ }, 360^{\circ })$	$30^{\circ }$

As $\Omega _0$ is varied, the long-lifetime orbit and its surrounding oscillatory structure in Fig. 1a–c shifts to impact the associated perilune groundtrack. With a shift that is predominantly in $q_{mean}$, the location of the long-lifetime orbit in the *pq*-plane falls into one of the following three cases: (1) average ω near $90^{\circ }$, (2) average ω near $270^{\circ }$, or (3) average *e* near 0. In Fig. 1d–f, the perilune groundtracks of the long-lifetime orbits at each value of $\Omega _0$ are plotted. These three perilune sequences produce groundtracks with distinct geometries, encircling d) the north pole or e) the south pole twice per revolution, or f) both hemispheres once per revolution. In addition, note that the shift in the oscillatory structure due to varying $\Omega _0$ can also influence which initial conditions lead to lunar impact and/or their duration.

## Results and discussion

The presented technical approach is used to generate a low lunar trajectory dataset. These trajectories are then clustered to produce a global cluster summary of geometrically distinct perilune sequences in the *pq*-plane that supports exploratory analysis. Representative trajectories are extracted from global clusters with perilune sequences that follow paths with a low drift in the *pq*-plane over 180 days. Each set is organized to possess a single initial inclination, one or more initial semi-major axes, and a distinct geometry in both the *pq*-plane and perilune groundtrack. These sets of long-lifetime orbits are then examined and compared to existing analytical predictions or numerical analyses.

### Trajectory dataset

**Fig. 2:**
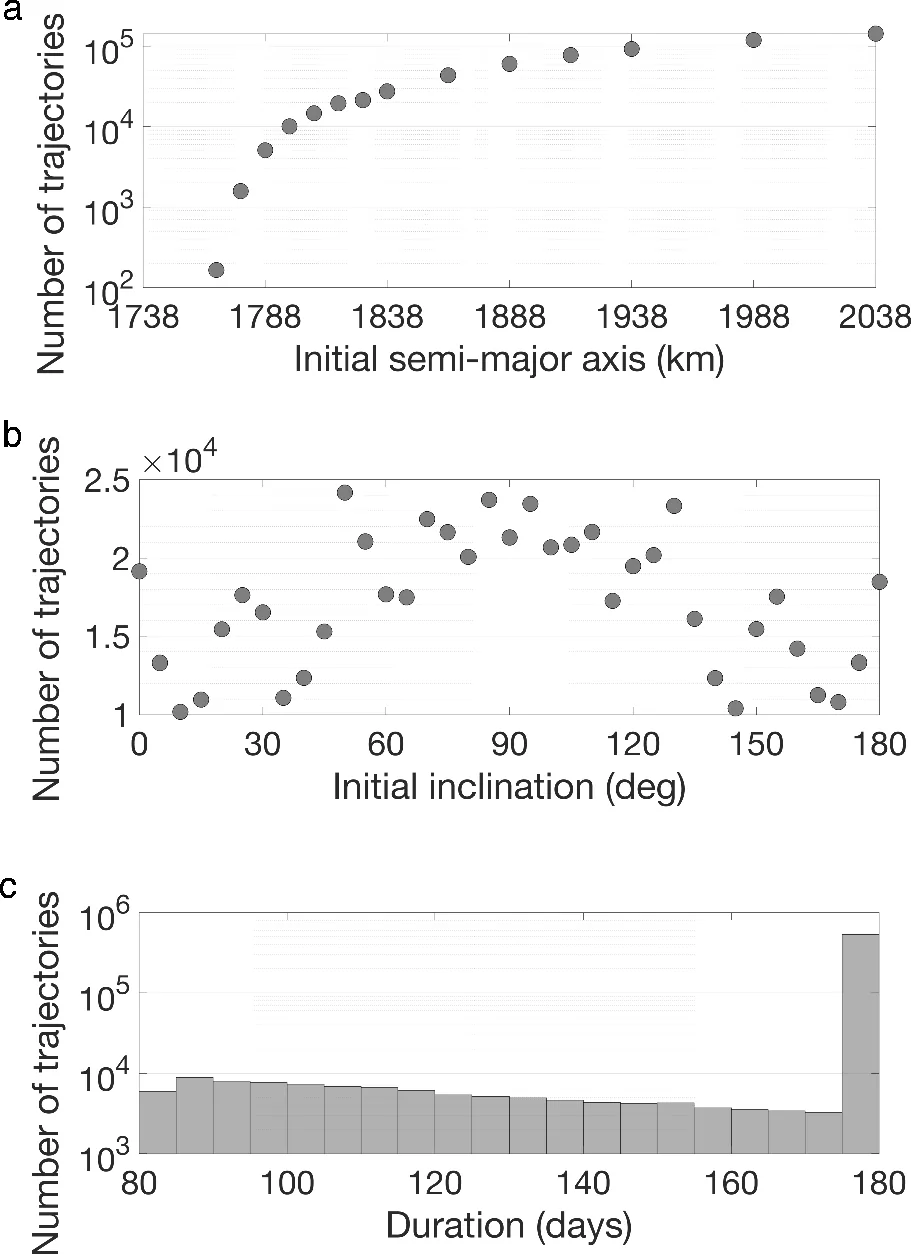
Summary of low lunar trajectories with a duration of at least three lunar rotational periods: number of trajectories at each, **a** initial semi-major axis and **b** initial inclination in the lunar principal axes, along with **c** a histogram of their duration

As described in Sect. 4.1, a low lunar trajectory dataset is generated from a sparsely sampled set of initial conditions. The selected ranges and step sizes of these quantities are listed in Table 1. From 1,144,755 sampled initial conditions, 638,181 low lunar trajectories possess durations exceeding three lunar rotational periods. Figure 2 summarizes the number of trajectories at each discretely sampled value of a) $a_0$ and b) $i_0$. In addition, Fig. 2c) displays a histogram of the flight times of trajectories across this dataset. These trajectories are equally distributed across 64 partitions that are composed of 9,972 members in all but the final partition.

### Clustering results

**Fig. 3:**
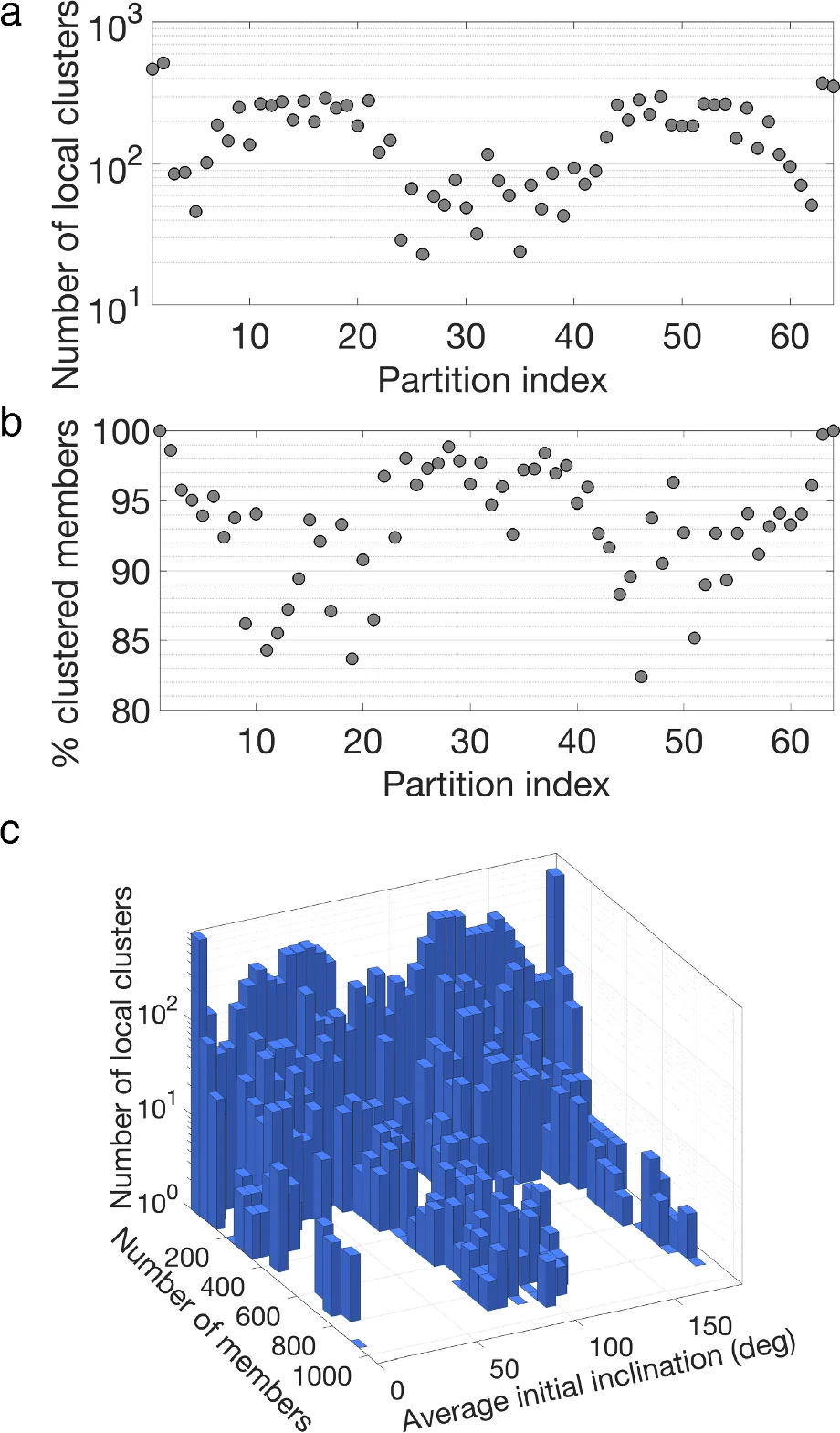
**a** Number of local clusters and **b** percentage of perilune sequences assigned to a cluster within each partition, and **c** two-dimensional histogram summarizing the distribution of local clusters as a function of the membership size and average initial inclination

Across all 64 partitions, 10,804 local clusters are computed from 93.31% of the dataset assigned to a cluster. Figure 3 displays a summary of a) the number of clusters on a logarithmic scale and b) the percentage of perilune sequences assigned to a cluster across each partition. Recall that the inclination increases from $0^{\circ }$ to $180^{\circ }$ from the first to last partition. In Fig. 3a and b, there are similar patterns in the number of clusters and clustered trajectories around partitions containing perilune sequences with an initial inclination of $90^{\circ }$; this pattern is analyzed later. Furthermore, in Fig. 3a, a larger number of clusters is recovered in the first two and last two partitions, where the inclination is close to $0^{\circ }$ or $180^{\circ }$. Many of these clusters encompass a small number of perilune sequences that exist in localized regions in $\boldsymbol{f}_{\dot{p}\dot{q}}$. This observation indicates sensitivity in the shape of the nearly planar path in *pq*-coordinates, possibly due to ω and Ω becoming undefined when $i=0^{\circ }$ or $i=180^{\circ }$.

Figure 3c displays a two-dimensional histogram summarizing the number of local clusters as a function of the membership size, with bin widths of 50, and average initial inclination, with bin widths of 5 degrees. Due to the large number of local clusters, this figure is only used to observe general trends. For instance, the majority of local clusters are small, consistent with the sparse sampling of the initial conditions and selected parameters governing the clustering process. However, larger clusters tend to exist for nearly planar orbits and those with a high average initial inclination between $70^\circ$ and $110^\circ$.

**Fig. 4:**
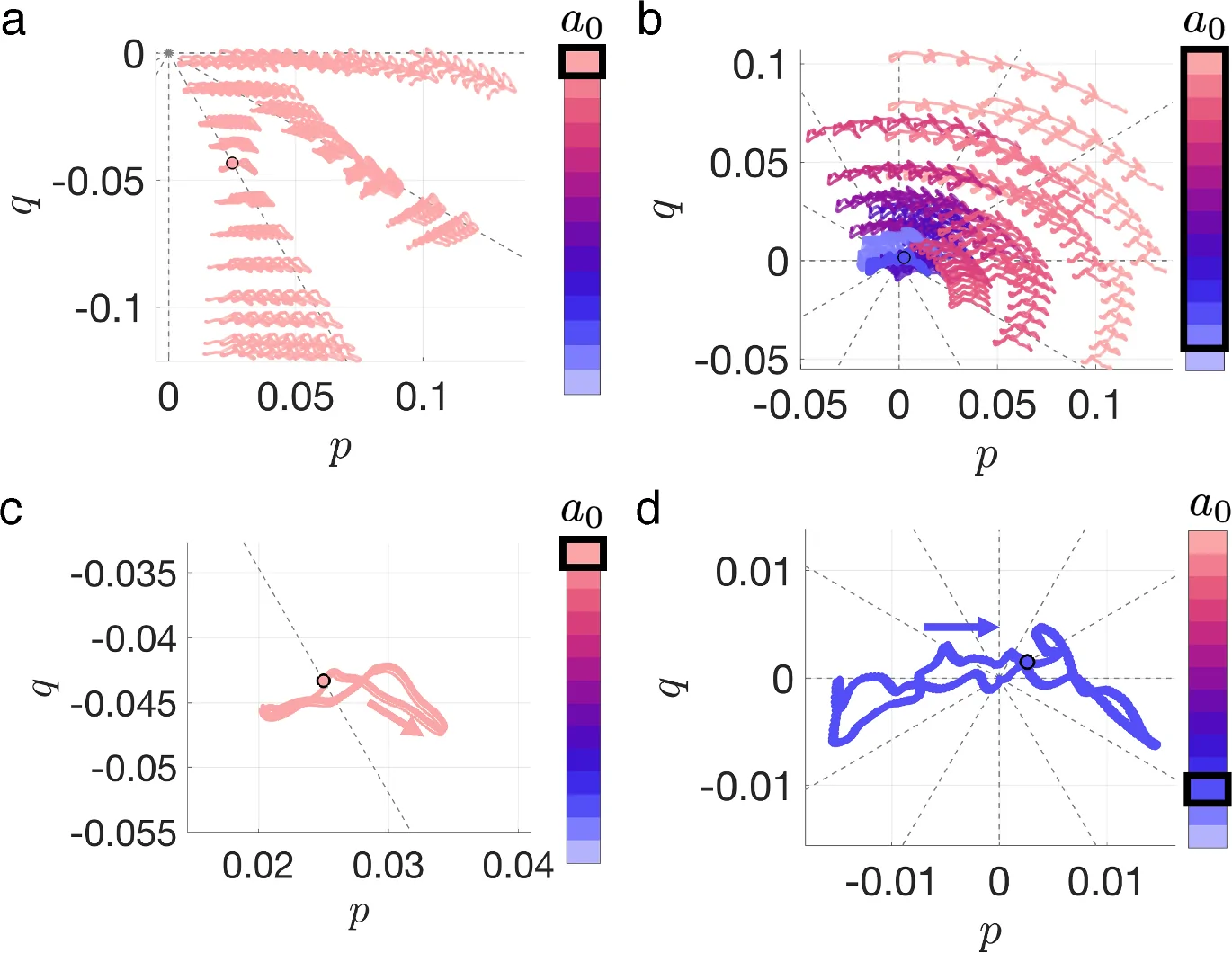
Selected local clusters from the 23rd partition in each column: **a**, **b** subset of 26 perilune sequences in the *pq*-plane, **c**, **d** cluster representatives in the *pq*-plane

**Fig. 5:**
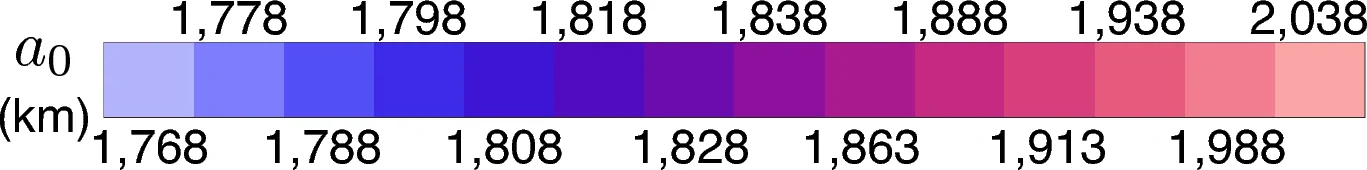
Mapping from initial semi-major axis to colors used for plotting perilune sequences

To demonstrate the clustering process and visualization, consider two local clusters from the 23rd partition. In Fig. 4a and b, a subset of 26 perilune sequences from these local clusters are plotted in the *pq*-plane and colored by $a_0$. The nonuniform mapping between $a_0$ and each color is defined in Fig. 5. Furthermore, the values of $a_0$ across all cluster members are highlighted by a black box on the colorbar to the right of Fig. 4a and b. Figure 4c and d displays a zoomed-in view of the cluster representatives, with their initial state highlighted by a black circle, associated value of $a_0$ highlighted on the colorbar, and the colored arrow identifying the direction of perilune evolution. Within each cluster, the grouped perilune sequences possess a similar shape within each revolution in the *pq*-plane and initial phasing. However, their initial *pq*-coordinates and drift evolve across the cluster. Furthermore, perilune sequences in distinct clusters possess a distinct shape. Accordingly, the clustering process successfully groups perilune sequences by their geometry in the *pq*-plane.

The cluster aggregation procedure is applied to all $K_{\ell }=10,804$ local clusters generated in Step 3 to produce a global cluster summary. In the first aggregation step, the local clusters are merged into 6,115 groups, each composed of perilune sequences with a similar shape and phasing in the *pq*-plane. In the second aggregation step, 1,247 groups are formed, each composed of perilune sequences with a similar shape in the *pq*-plane, independent of initial phasing. Combining the two merging decisions produces a total of $K_{g}=885$ global clusters that summarize the 638,181 low lunar trajectories.

**Fig. 6:**
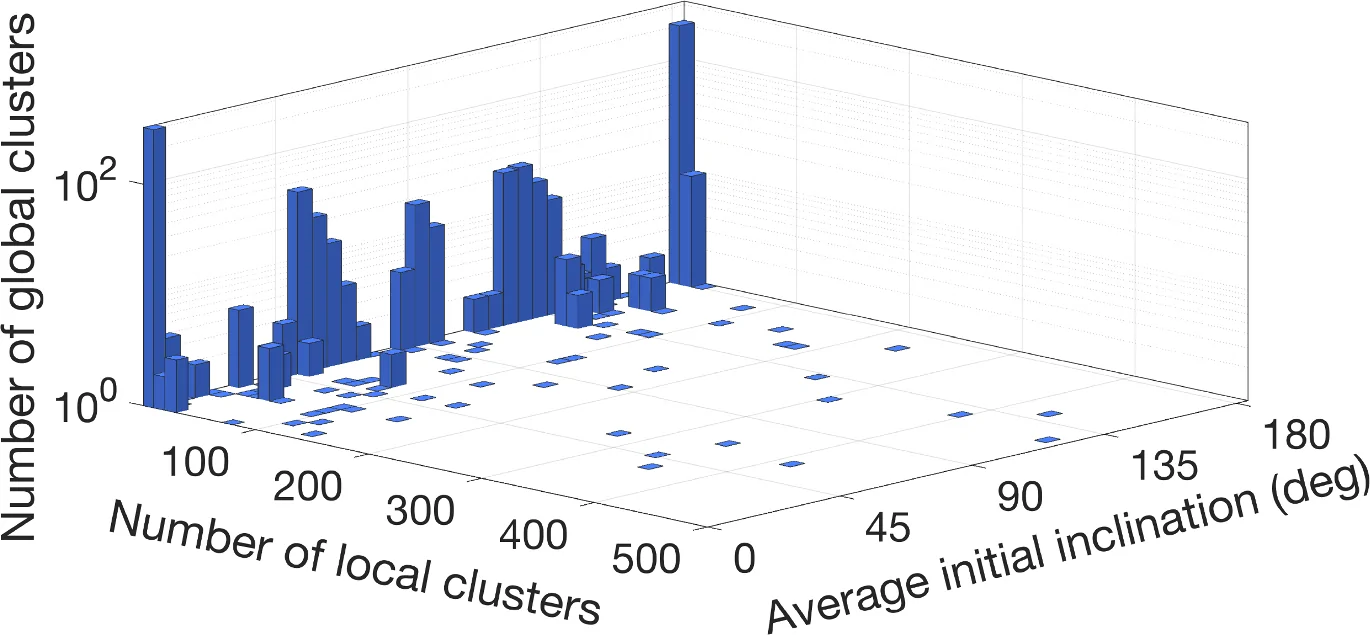
Two-dimensional histogram displaying the number of global clusters that are formed by merging various numbers of local clusters with various average inclinations

A summary of the global clusters appears in the two-dimensional histogram in Fig. 6. This figure displays the distribution of global clusters as a function of the number of merged local clusters and the average inclination, with bin widths of 10 and $5^{\circ }$, respectively. A small number of these global clusters are formed by merging a large number of local clusters, typically including perilune sequences of long-lifetime orbits. However, for nearly planar low lunar trajectories, global clusters tend to be formed by individual local clusters or a small number of merged local clusters. This lack of aggregation is due to sensitivity of the shape of their perilune sequence in the *pq*-plane.

### Long-lifetime trajectories

**Fig. 7:**
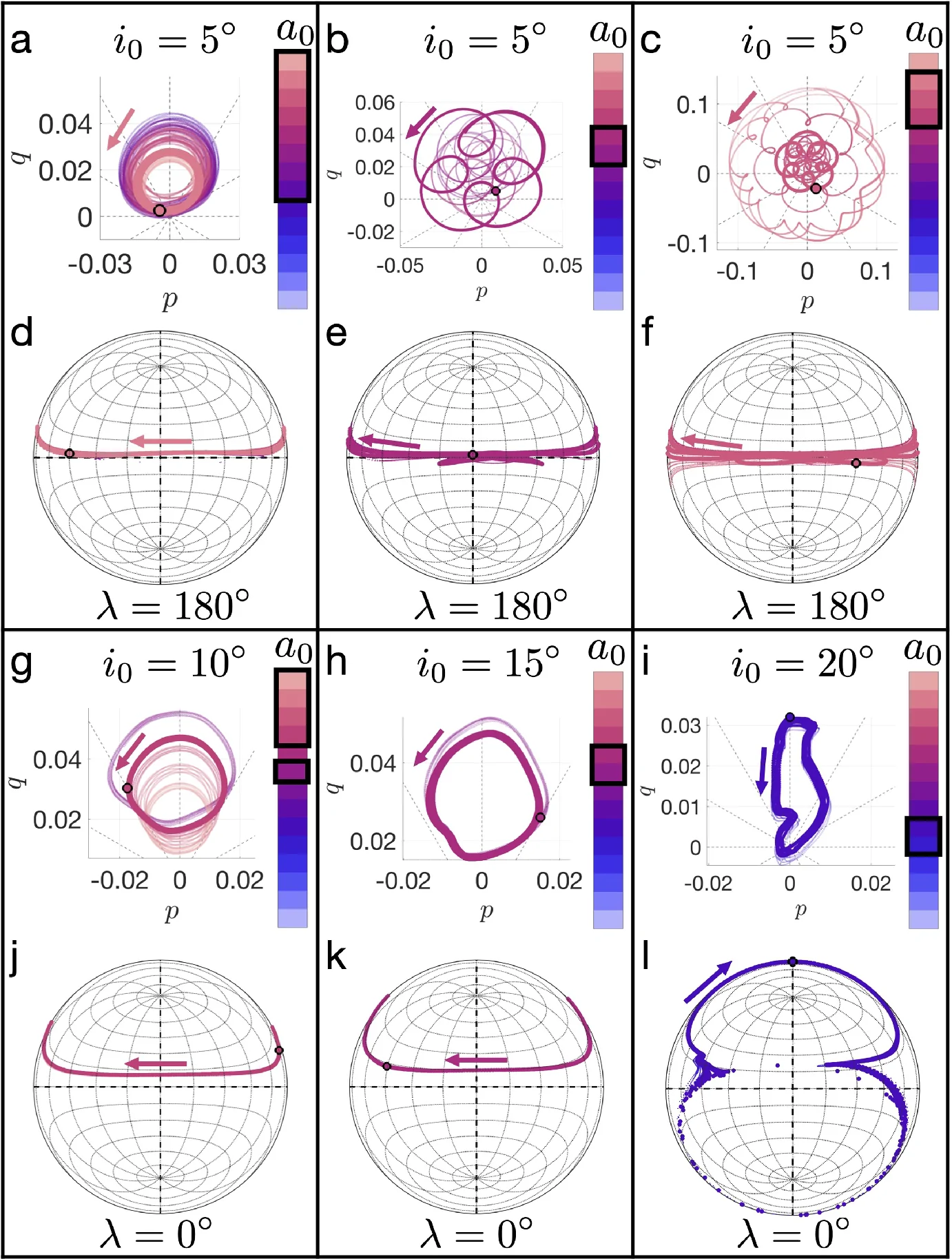
Perilune sequences of long-lifetime orbits, depicted **a**–**c**, **g**–**i** in the *pq* plane and **d**–**f**, **j**–**l** projected onto the Moon; $a_0$ of plotted trajectories highlighted on colorbar as defined in Fig. 5

**Fig. 8:**
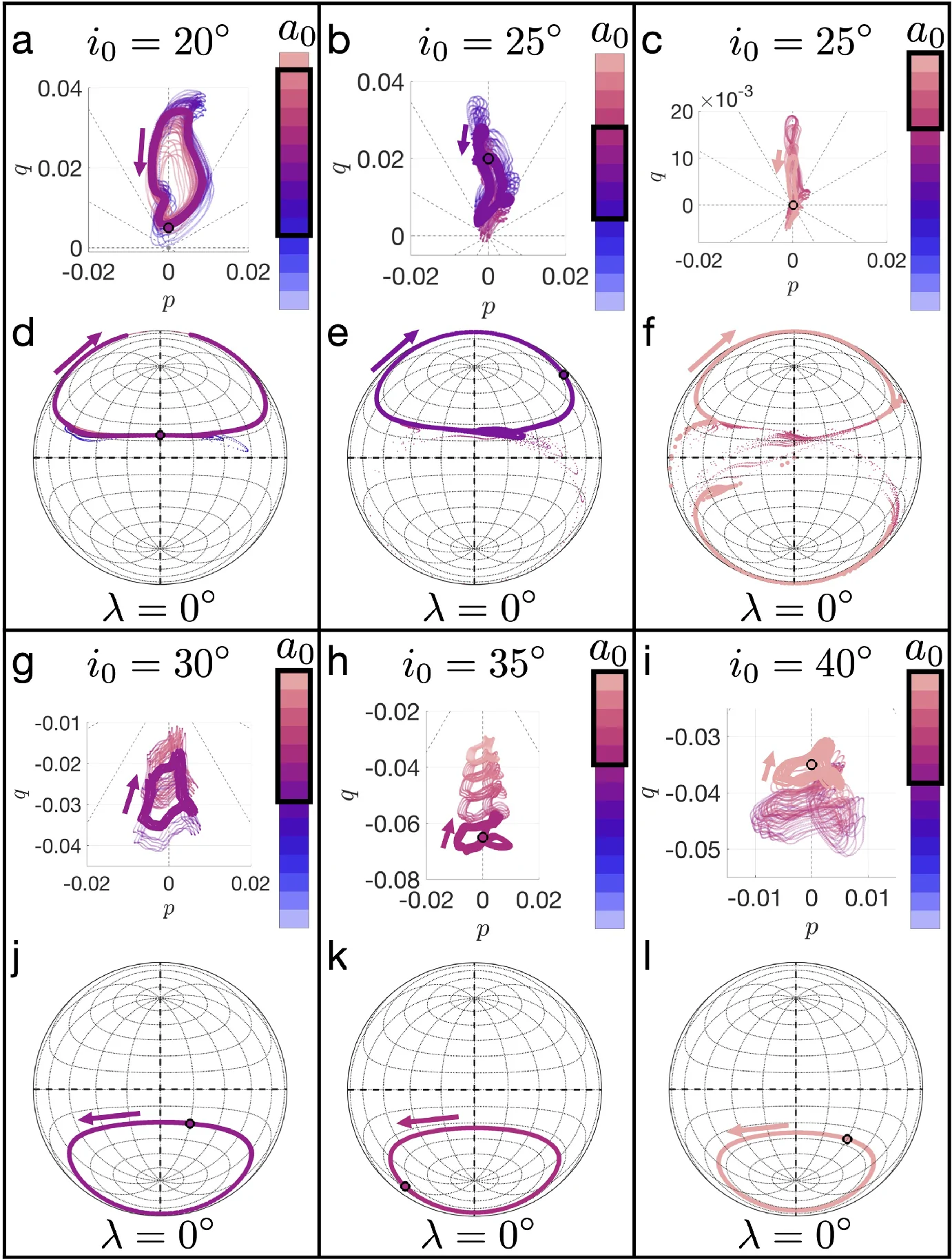
Perilune sequences of long-lifetime orbits, depicted **a**–**c**, **g**–**i** in the *pq* plane and **d**–**f**, **j**–**l** projected onto the Moon; $a_0$ of plotted trajectories highlighted on colorbar as defined in Fig. 5

**Fig. 9:**
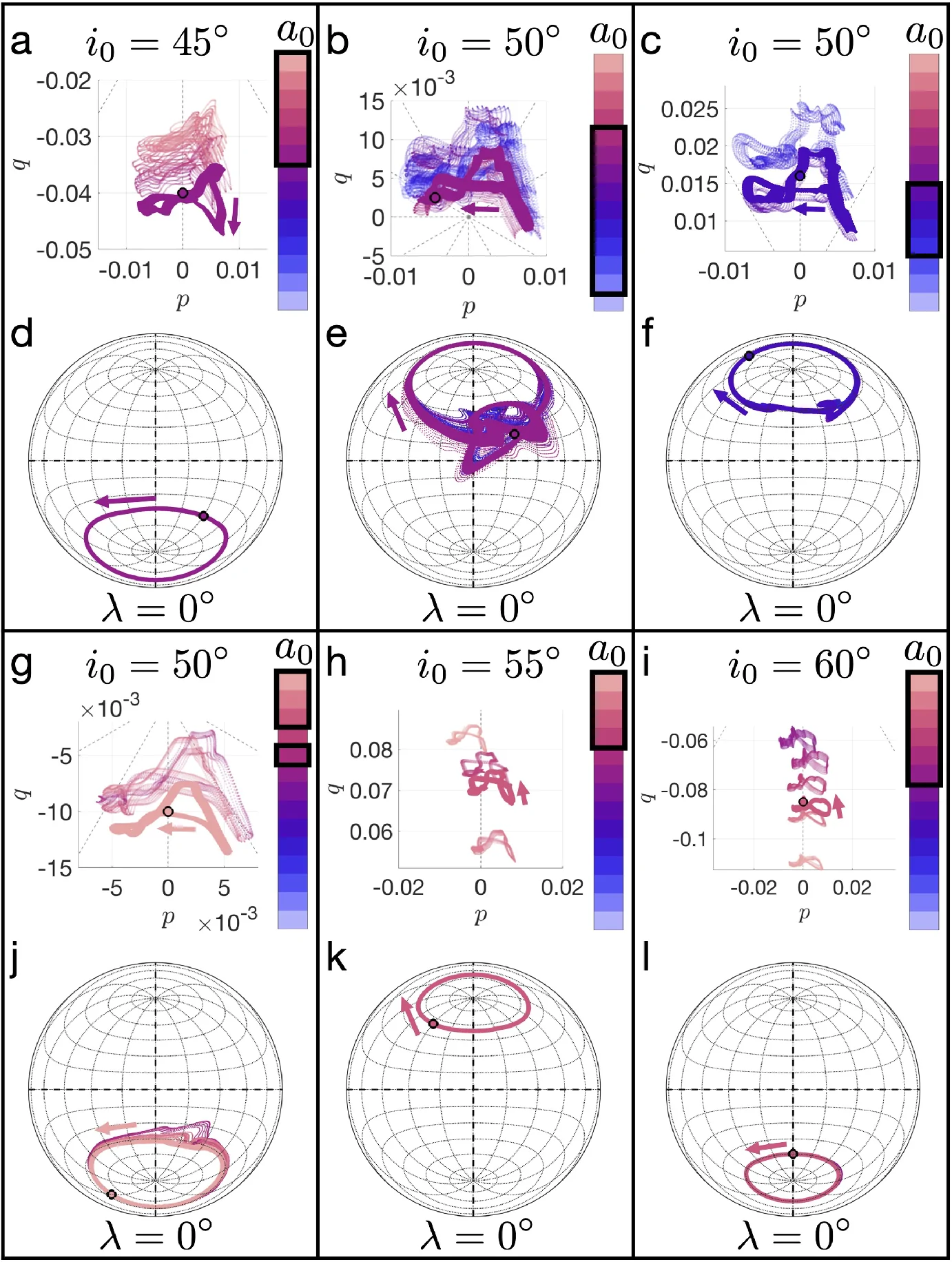
Perilune sequences of long-lifetime orbits, depicted **a**–**c**, **g**–**i** in the *pq* plane and **d**–**f**, **j**–**l** projected onto the Moon; $a_0$ of plotted trajectories highlighted on colorbar as defined in Fig. 5

**Fig. 10:**
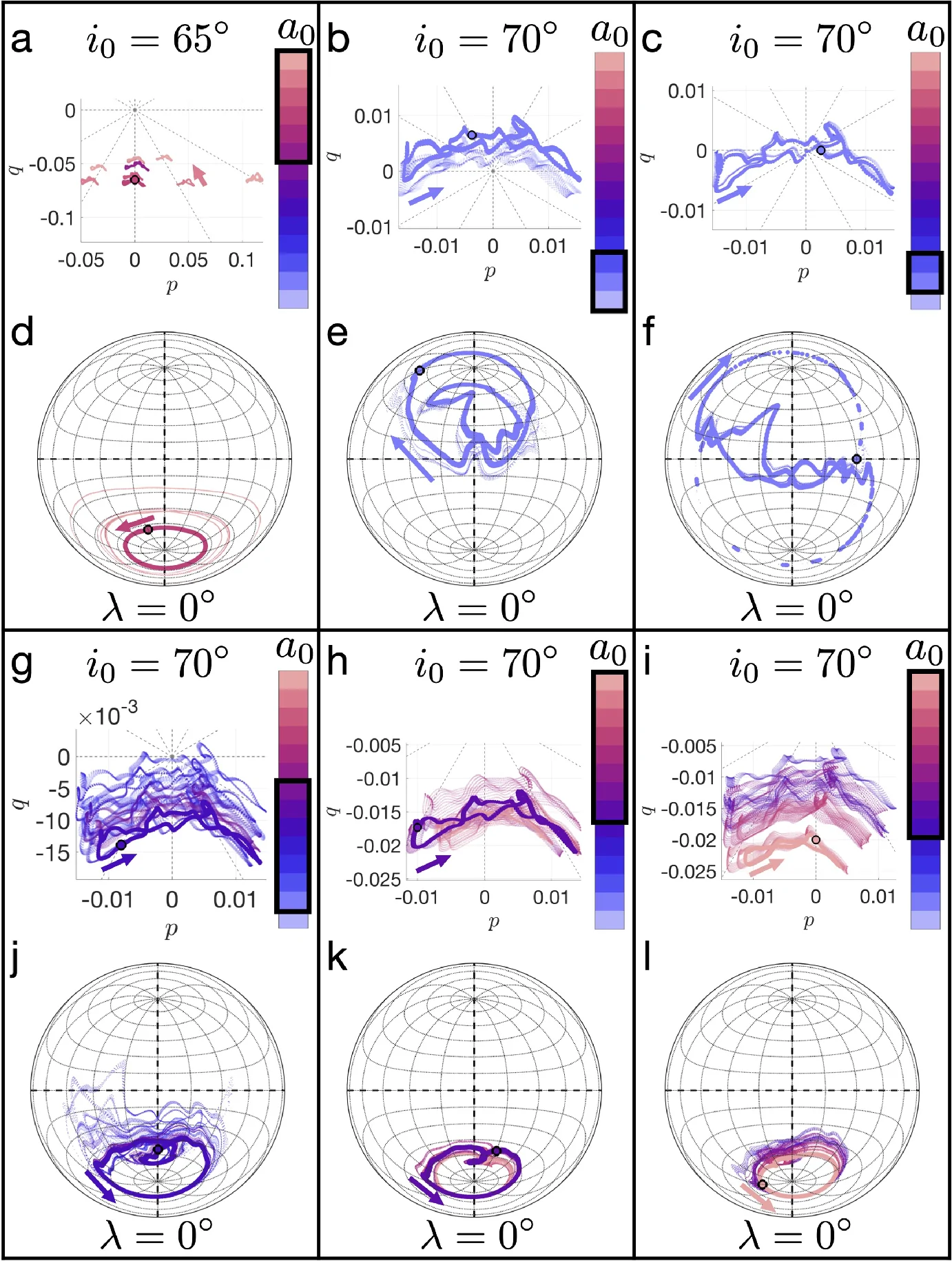
Perilune sequences of long-lifetime orbits, depicted **a**–**c**, **g**–**i** in the *pq* plane and **d**–**f**, **j**–**l** projected onto the Moon; $a_0$ of plotted trajectories highlighted on colorbar as defined in Fig. 5

**Fig. 11:**
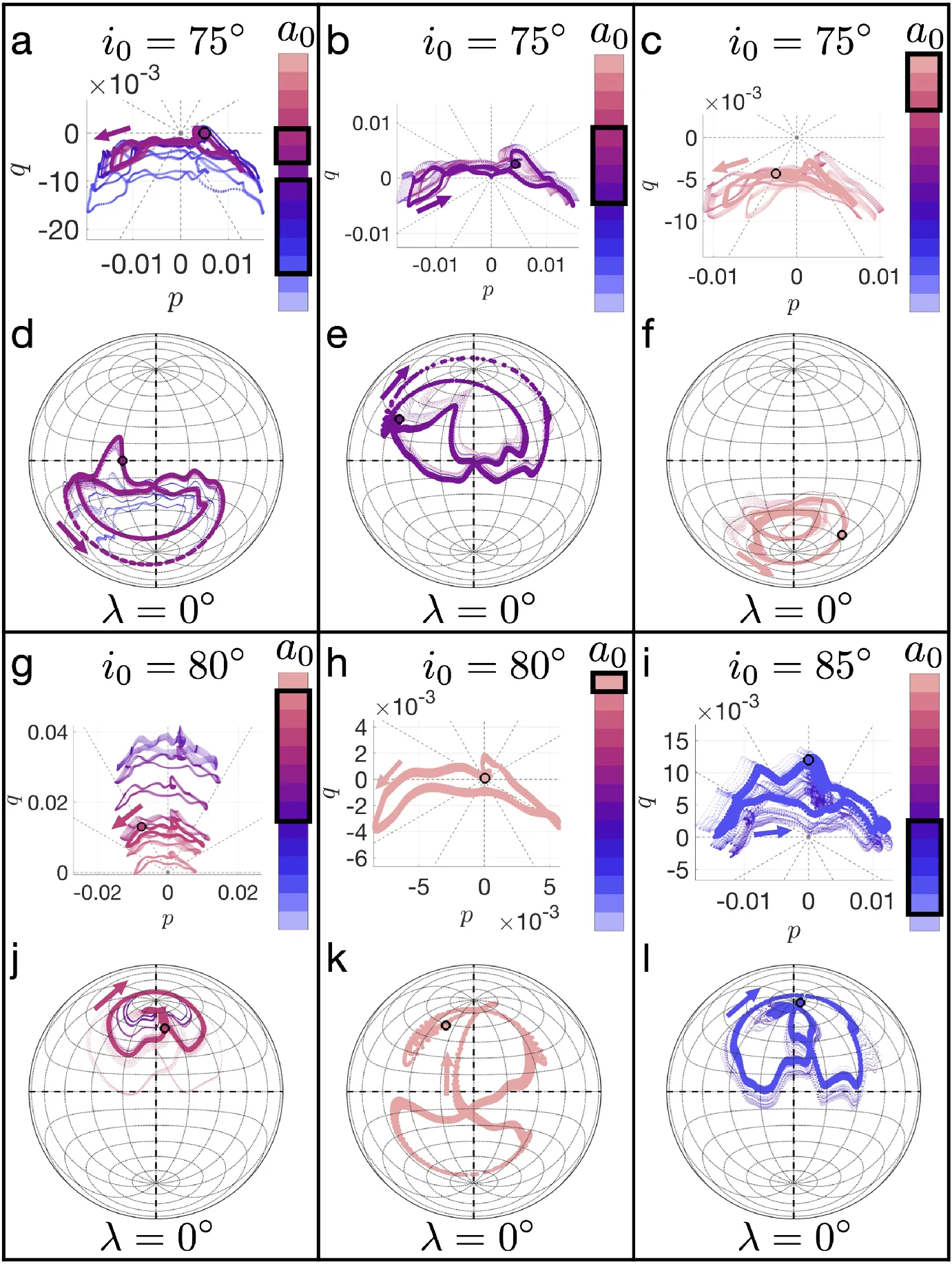
Perilune sequences of long-lifetime orbits, depicted **a**–**c**, **g**–**i** in the *pq* plane and **d**–**f**, **j**–**l** projected onto the Moon; $a_0$ of plotted trajectories highlighted on colorbar as defined in Fig. 5

**Fig. 12:**
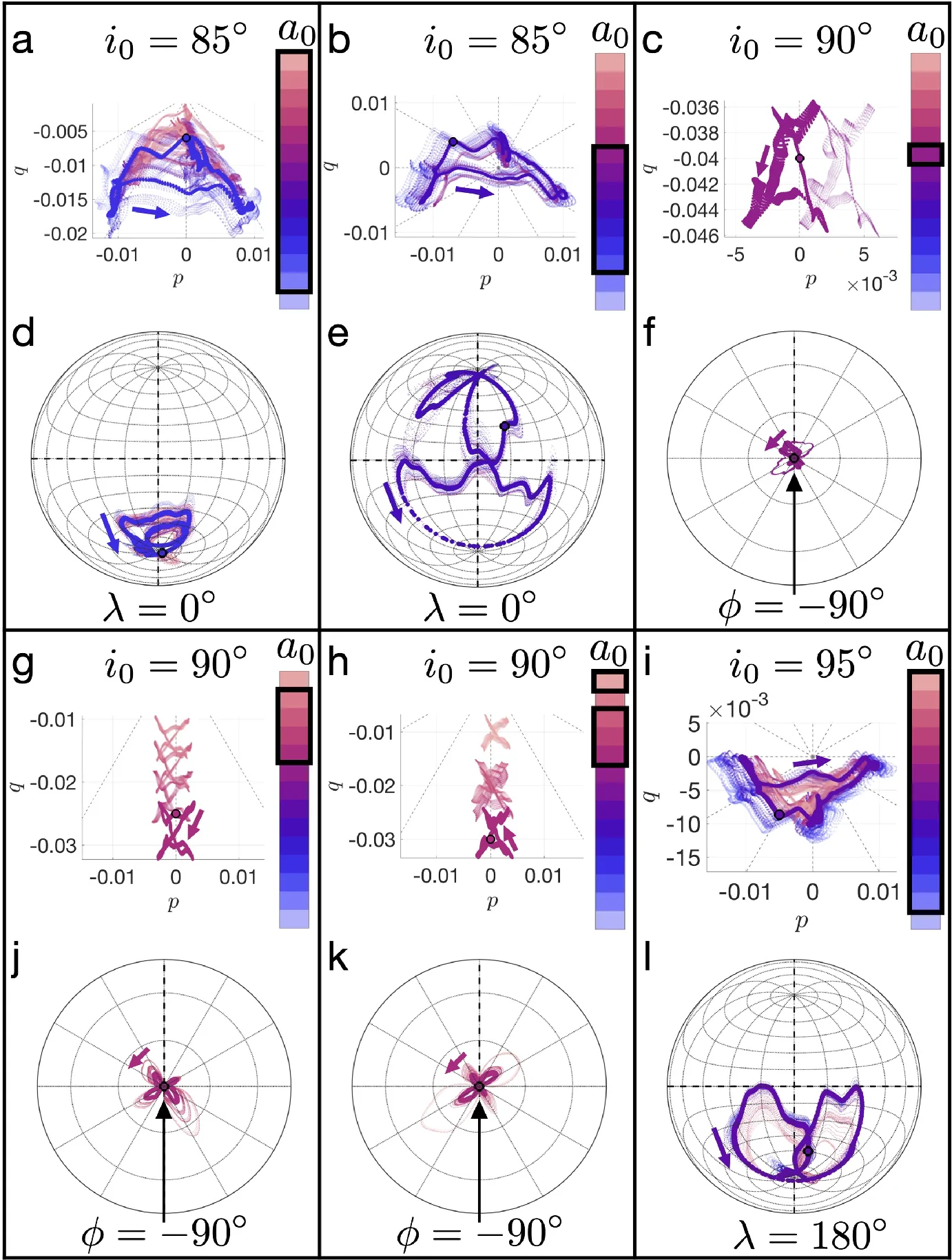
Perilune sequences of long-lifetime orbits, depicted **a**–**c**, **g**–**i** in the *pq* plane and **d**–**f**, **j**–**l** projected onto the Moon; $a_0$ of plotted trajectories highlighted on colorbar as defined in Fig. 5

**Fig. 13:**
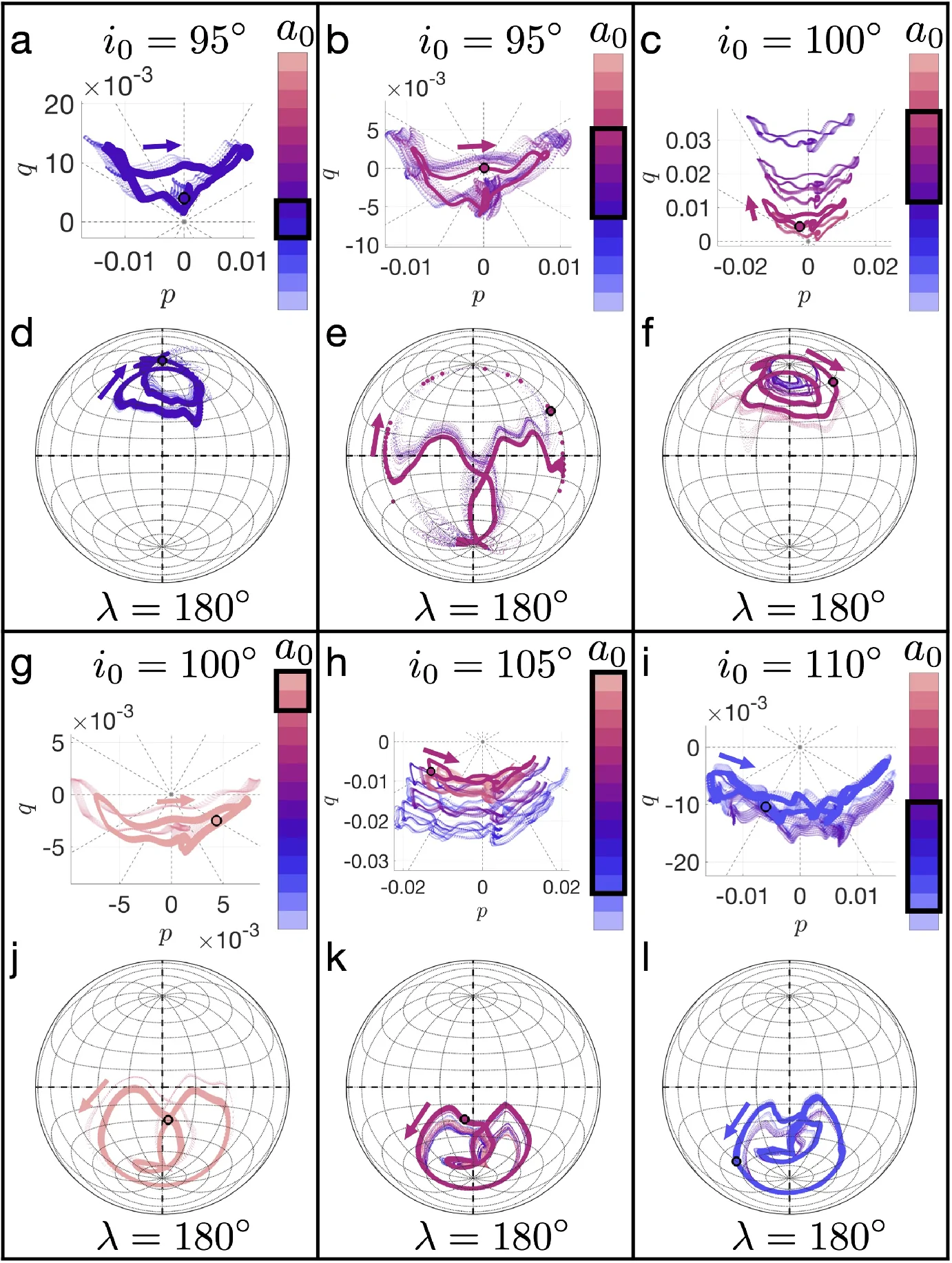
Perilune sequences of long-lifetime orbits, depicted **a**–**c**, **g**–**i** in the *pq* plane and **d**–**f**, **j**–**l** projected onto the Moon; $a_0$ of plotted trajectories highlighted on colorbar as defined in Fig. 5

**Fig. 14:**
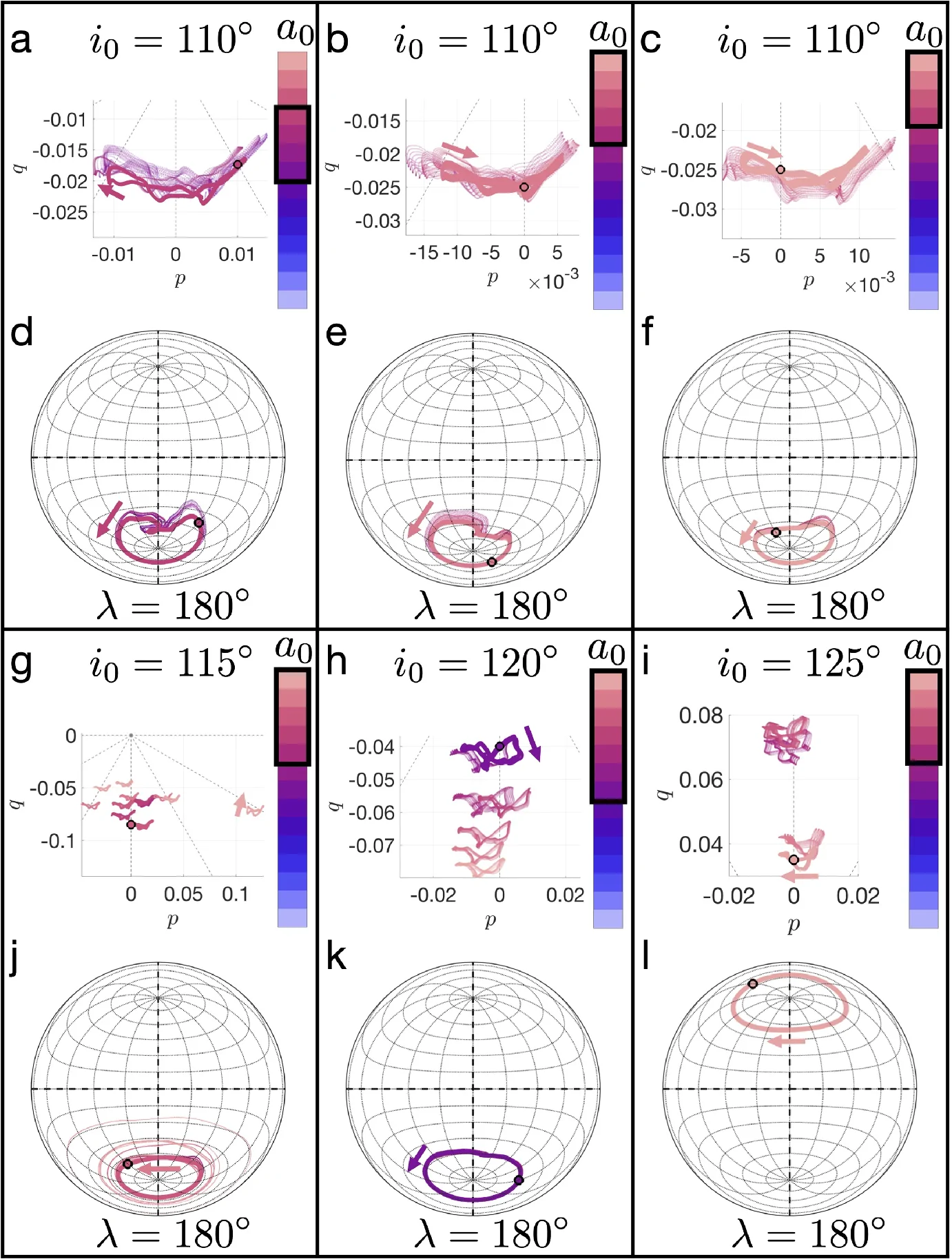
Perilune sequences of long-lifetime orbits, depicted **a**–**c**, **g**–**i** in the *pq* plane and **d**–**f**, **j**–**l** projected onto the surface of the Moon; colorbars depict $a_0$ of selected trajectories as defined in Fig. 5

**Fig. 15:**
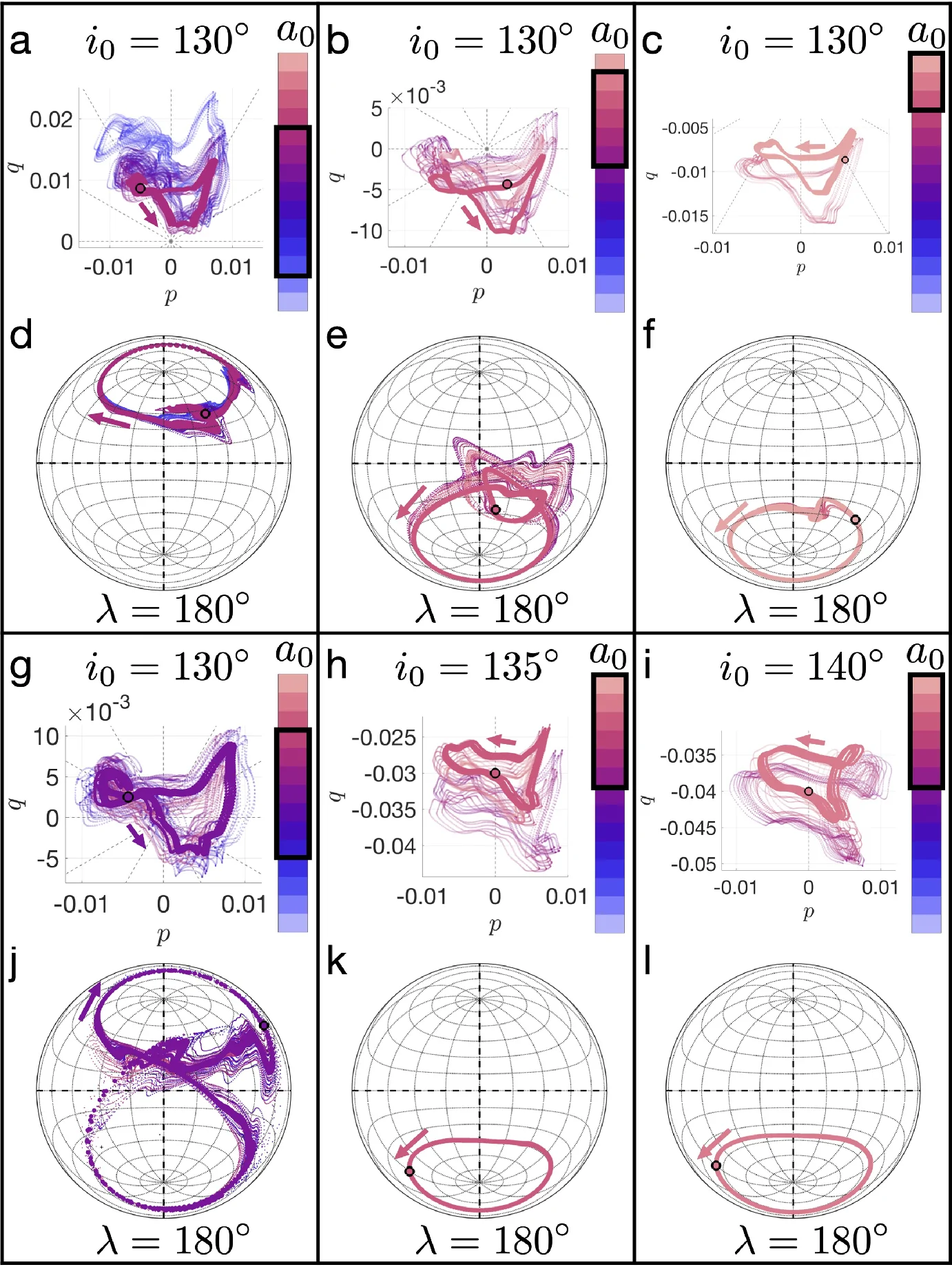
Perilune sequences of long-lifetime orbits, depicted **a**–**c**, **g**–**i** in the *pq* plane and **d**–**f**, **j**–**l** projected onto the Moon; $a_0$ of plotted trajectories highlighted on colorbar as defined in Fig. 5

**Fig. 16:**
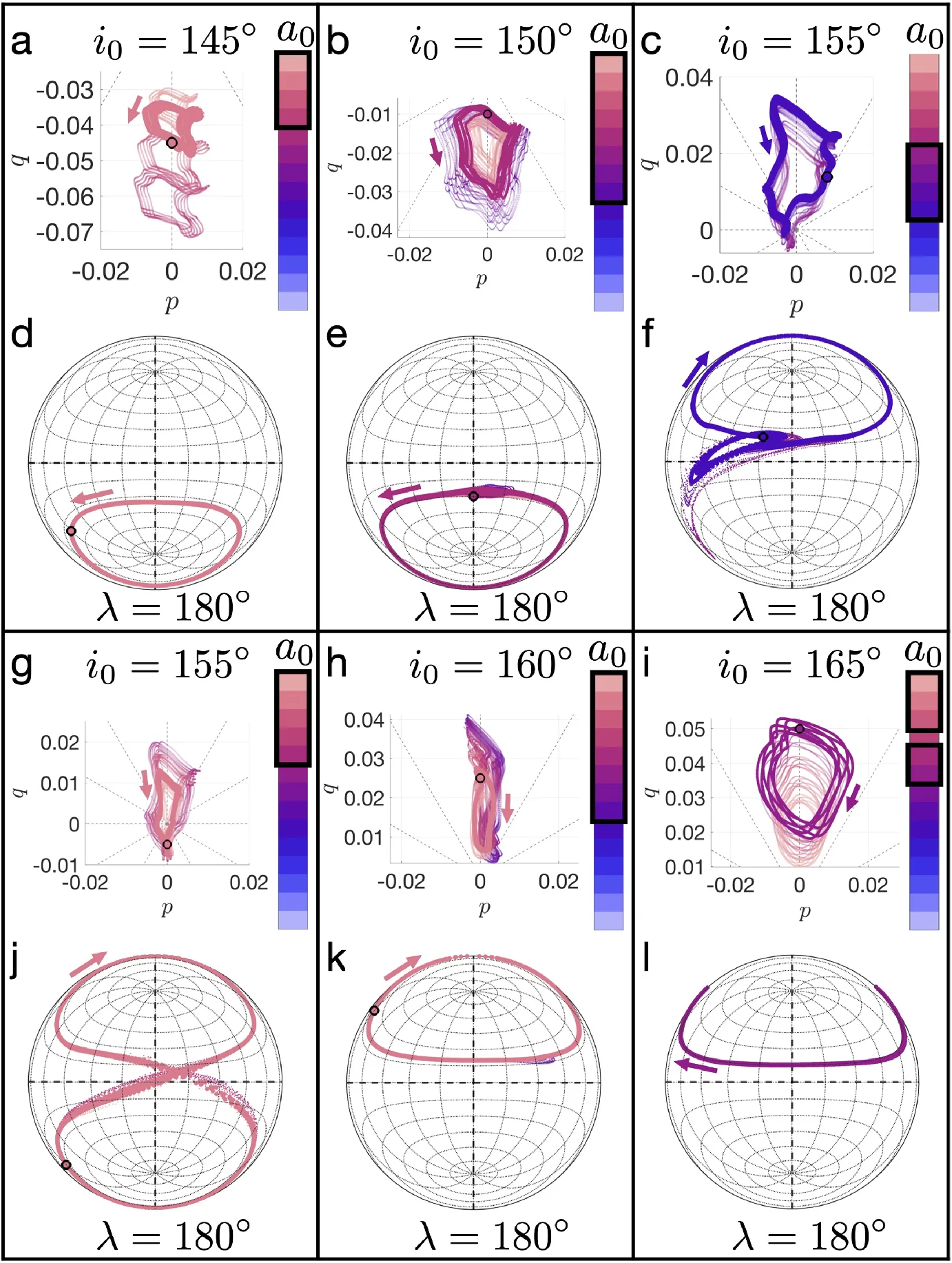
Perilune sequences of long-lifetime orbits, depicted **a**–**c**, **g**–**i** in the *pq* plane and **d**–**f**, **j**–**l** projected onto the Moon; $a_0$ of plotted trajectories highlighted on colorbar as defined in Fig. 5

**Fig. 17:**
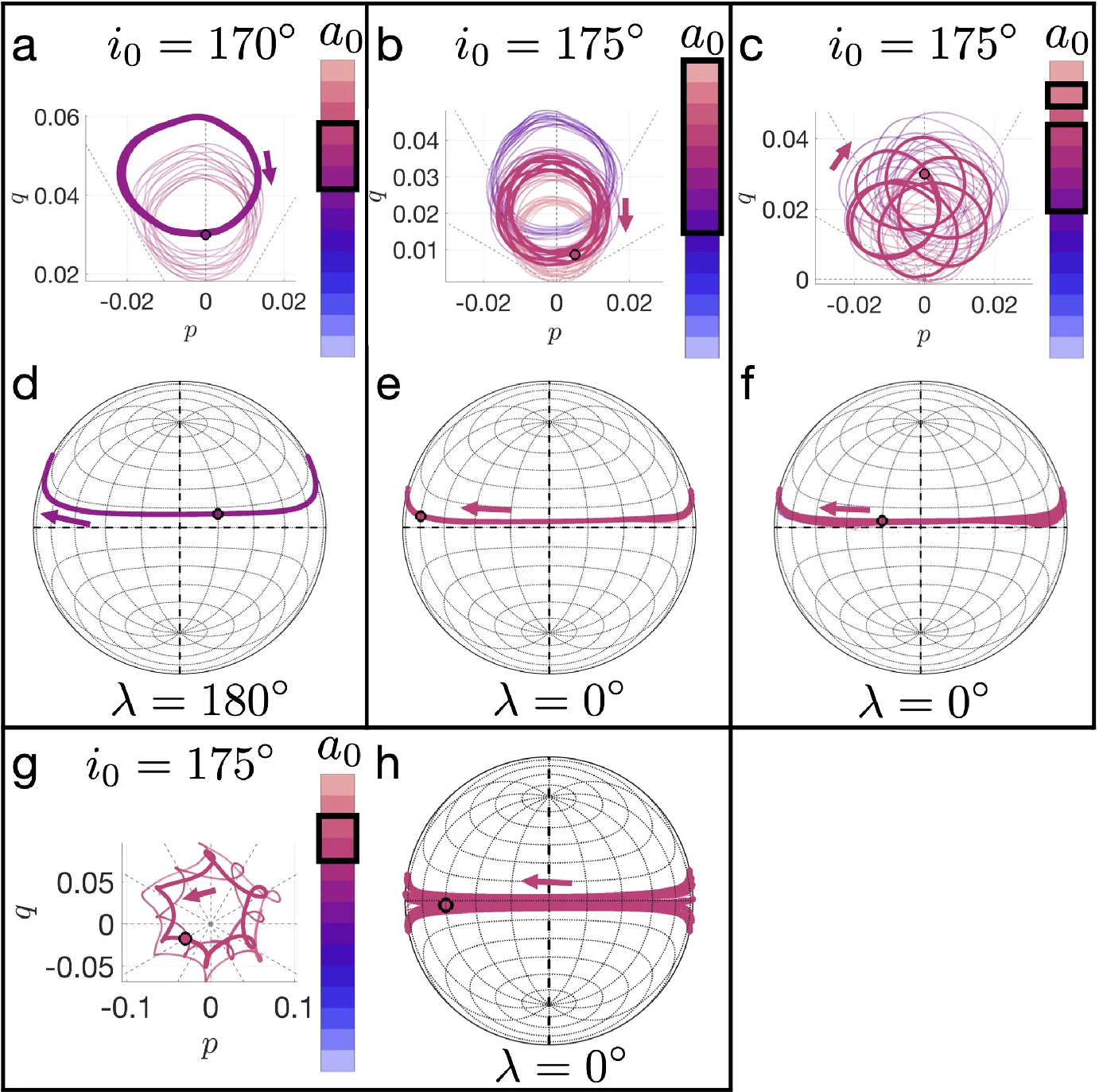
Perilune sequences of long-lifetime orbits, depicted **a**–**c**, **g** in the *pq* plane and **d**–**f**, **h** projected onto the Moon; $a_0$ of plotted trajectories highlighted on colorbar as defined in Fig. 5

**Fig. 18:**
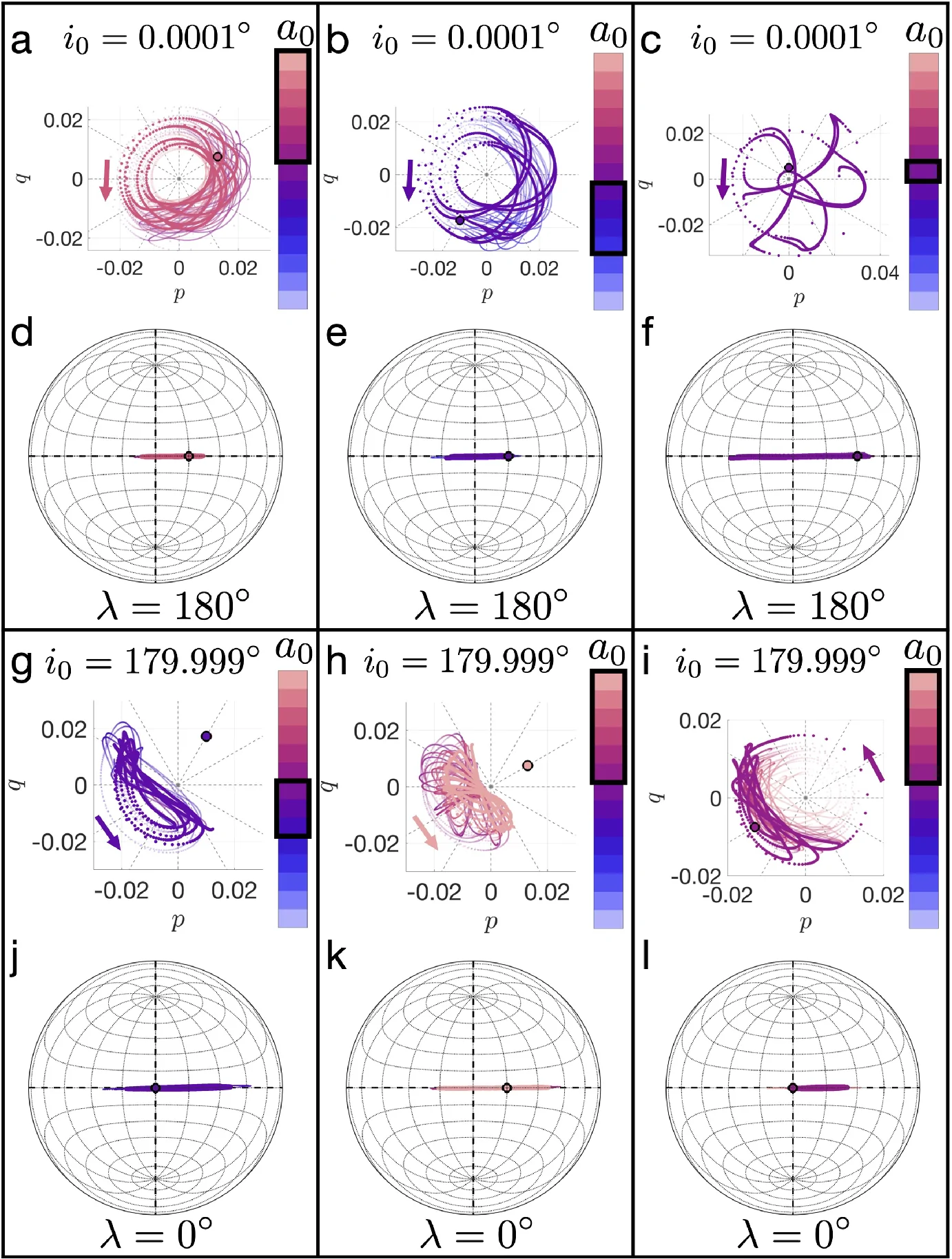
Perilune sequences of long-lifetime orbits, depicted **a**–**c**, **g**–**i** in the *pq* plane and **d**–**f**, **j**–**l** projected onto the Moon; $a_0$ of plotted trajectories highlighted on colorbar as defined in Fig. 5

The clustering results are used to manually identify sets of low lunar, long-lifetime trajectories producing perilune sequences with geometrically similar paths in the *pq*-plane and groundtracks. Figures 7, 8, 9, 10, 11, 12, 13, 14, 15, 16 and 17 display long-lifetime orbits with initial inclinations that increase across the range $5^{\circ } \le i_0 \le 175^{\circ }$. Figure 18 displays selected, near-planar, long-lifetime orbits. In each figure, up to six sets of long-lifetime orbits are displayed. The first row, labeled a)-c), and third row, labeled g)-i), contain plots of perilune sequences in the *pq*-plane. Each of these subfigures is isolated to a single initial inclination and contains one or more perilune sequences at each applicable initial semi-major axis with a sufficiently similar groundtrack. The associated values of $a_0$ are indicated by the black box around segments of the nonuniform colorbar on the right, defined in Fig. 5. Additional long-lifetime orbits may exist at other values of $a_0$ but might not be captured in these figures due to the sparsity of the dataset. Nevertheless, the perilune groundtracks are plotted immediately below or to the right of each path in the *pq*-plane. Although both subfigures depict useful information, specific perilune sequences are identified in the remaining text by the subfigure displaying their path in the *pq*-plane for brevity.

The initial orbital elements and global cluster assignment of each plotted perilune sequence is provided in the form of a text file (Online Resource 1). To generate trajectories from this data, the dynamical model, kernel files, and propagation interval described in Sect. 2 should be used. Note, however, that different numerical implementations may produce slightly different results when propagating trajectories over long durations. Furthermore, trajectories are only generated for up to 180 days in this paper. Some, but not necessarily all, trajectories may exist near orbits that maintain boundedness or a low drift over much longer durations.

Within each subfigure, a single set of long-lifetime orbits is extracted from one or more clusters that exist at a single inclination, except for nearly planar orbits. When $i_0 \le 15^{\circ }$ or $i_0 \ge 165^{\circ }$, the shape of the perilune sequences associated with long-lifetime orbits in the *pq*-plane evolves slowly as a function of inclination. Accordingly, Fig. 7a, b, c, g, and h displays perilune sequences from the same global cluster. Similarly, Figs. 16i and 17a, b, c, and g also display perilune sequences from the same global cluster. Most of the remaining sets of long-lifetime orbits are generated from a single cluster that exists at one initial inclination for $15^{\circ }< i_0 < 165^{\circ }$. However, at initial inclinations such as $i_0=70^{\circ }$, a single cluster includes members depicted across five subfigures in Fig. 10b, c, g, h, and i because they possess a similar shape in the *pq*-plane, but distinct groundtrack geometries as the perilune sequence revolves above, below, or around the origin of the *pq*-plane. Alternatively, at $i_0=50^{\circ }$ and $i_0=130^{\circ }$, for example, the perilune sequences displayed within Figs. 9b, c, and g and 15a, b, c, and g are separated across many clusters but manually grouped into the same plot. The separation is consistent with the clustering process being sensitive to substantial local variations in the shape within the *pq*-plane. Nevertheless, these observations suggest that the shape of the perilune sequences in the *pq*-plane is sensitive to initial inclination.

As the initial semi-major axis evolves along a set of long-lifetime orbits depicted in each pair of subfigures, the path traced out by the perilune sequence in the *pq*-plane as well as its groundtrack gradually varies. In most sets, the perilune sequences tend to shift closer to the $\omega =0^{\circ }$ and $\omega =180^{\circ }$ lines in the *pq*-plane as $a_0$ increases, indicating that the mean eccentricity decreases. Exceptions to this observation include selected long-lifetime orbits at $i_0\in \{5^{\circ },50^{\circ },55^{\circ },60^{\circ }, 70^{\circ }, 110^{\circ }, 120^{\circ }, 125^{\circ },175^{\circ }\}$. The shift in the *pq*-plane as $a_0$ evolves is consistent with existing geometrical interpretations from secular perturbation theory. Specifically, the instantaneous eccentricity vector can be expressed in terms of two vectors: (1) a ‘forced’ component that reflects the secular evolution and evolves as the semi-major axis changes, and (2) a ‘free’ component that oscillates around the ‘forced’ component and is derived from the boundary conditions Murray and Dermott (1999).

Some sets of long-lifetime orbits that exist at neighboring values of the initial inclination exhibit similar geometries. To demonstrate this observation, Table 2 lists twelve manually identified groups of perilune sequences that exist across one or more nearby initial inclinations in the range $5^{\circ } \le i_0 \le 175^{\circ }$ but exhibit small differences in their shape in the *pq*-plane and groundtrack. For each group, the associated subfigures of the perilune sequences are identified along with a brief description of their shape in the *pq*-plane and groundtracks. For orbits with $i_0\ne 90^{\circ }$, the initial inclination values are listed in two sets or ranges: the first includes prograde orbits whereas the second includes retrograde orbits. It is possible that some sets that are isolated to distinct clusters or initial inclinations actually exist along continuous families, as discussed later in the comparison to existing analytical predictions. However, the sparsity of the generated dataset may limit the identification of this gradual geometric evolution.

The shape of the perilune sequences for prograde and retrograde orbits with the same difference in inclination from $i_0=90^{\circ }$ often exhibits anti-symmetry in the *pq*-plane. For example, consider the prograde long-lifetime orbits with $i_0\in [30^{\circ },45^{\circ }]$ in group 3, as depicted in Figs. 8g, h, i and 9a. The shape of their perilune sequences in the *pq*-plane is nearly anti-symmetric to the retrograde long-lifetime orbits with $i_0\in [135^{\circ },150^{\circ }]$, as displayed in Figs. 15h, i and 16a, b. However, across the two sets of long-lifetime orbits, their mean location in the *pq*-plane as well the range of associated initial semi-major axes is similar. Accordingly, their perilune groundtracks possess similar shapes. Alternatively, at low inclinations, the perilune sequences form resonant paths in the *pq*-plane. Along the prograde orbits, loops form interior to the path whereas along retrograde orbits, the loops form exterior to the path.

There are some cases where geometrically similar long-lifetime orbits are not recovered at symmetric inclinations around $i_0=90^{\circ }$. For example, at $i_0=70^{\circ }$, the perilune groundtracks in Fig. 10e revolve in the northern hemisphere and in Fig. 10f encircle both hemispheres. Perilune sequences with similar groundtracks are not recovered at $i_0=110^{\circ }$. Determining whether their absence is due to nonexistence, the dataset sparsity, their stability, or the clustering process, is a useful avenue of future work.

The perilune groundtracks tend to exhibit geometric characteristics in the lunar principal axes that are influenced by the initial inclination. For instance, multiple groups of perilune sequences listed in Table 2 produce groundtracks with knots that form over the $+\hat{X}$ hemisphere for prograde orbits and $-\hat{X}$ hemisphere for retrograde orbits. In contrast, at inclinations of $i_0 \le 5^{\circ }$ for prograde or $i_0 \ge 175^{\circ }$ for retrograde orbits, the perilune sequences form a knot near the $-\hat{X}$ or $+\hat{X}$ axis, respectively. Furthermore, for the selected near-planar long-lifetime orbits in Fig. 18, the perilune groundtracks are tightly bounded near these same axes. Further analysis across a broader range of dynamical models could reveal if these characteristics are due to the properties of the lunar gravitational field or the point mass gravitational interaction with the Earth, which is located predominantly in the $\hat{X}$ direction.

**Fig. 19:**
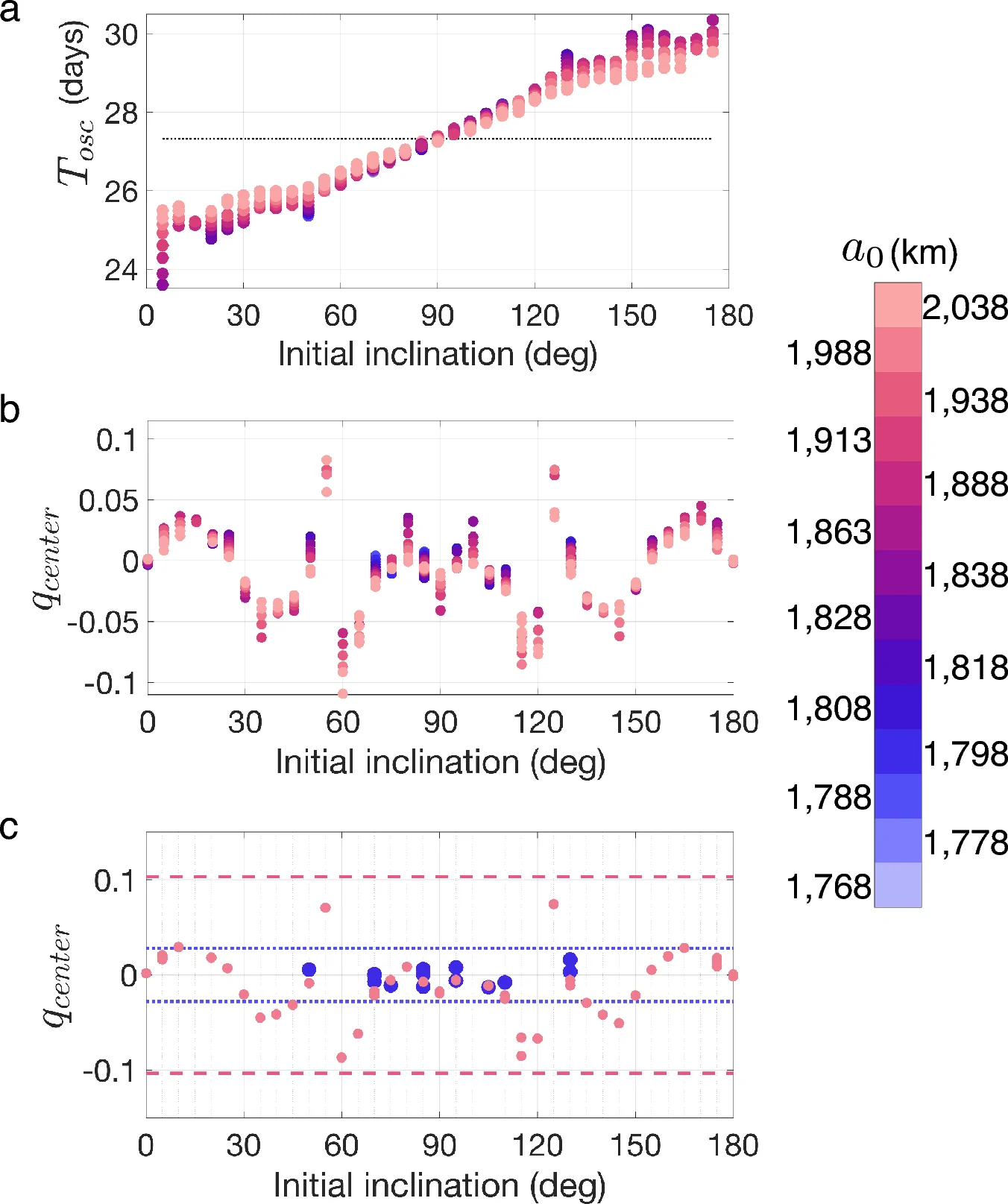
Characteristics of long-lifetime orbits across inclination: **a** period of revolution in the *pq*-plane, **b** mean value of *q* at all $a_0$, and **c** mean value of *q* at $a_0=1,788$ km and $a_0=1,938$ km

The perilune sequences of long-lifetime orbits trace out paths in the *pq*-plane with a period of revolution that varies as a function of initial inclination. The numerically estimated values of $T_{osc}$ for the nonresonant orbits with $5^{\circ } \le i_{0} \le 175^{\circ }$ are plotted in Fig. 19a as a function of initial inclination. Each point represents a single perilune sequence from Figs. 7, 8, 9, 10, 11, 12, 13, 14, 15, 16 and 17 and is colored by the associated initial semi-major axis. At $i_0=90^{\circ }$, the perilune sequences trace out one revolution in the *pq*-plane over one lunar rotational period. However, as the inclination evolves away from $90^{\circ }$, the value of $T_{osc}$ also evolves away from the lunar rotational period. Furthermore, as the initial semi-major axis decreases at each value of $i_0$, the value of $T_{osc}$ further evolves away from the lunar rotational period.

### Comparison to frozen orbits in the literature

#### Comparison to analytically predicted eccentricity and inclination across semi-major axis

The numerically generated long-lifetime orbits, with a low drift over 180 days, are compared with analytical predictions from Lara (2011) and, more recently, updated by Yarndley et al. (2020). Lara (2011) presented analytically derived families of eccentricity and inclination combinations that produce lunar frozen orbits with semi-major axes between 1,788 km and 1,938 km. The dynamical model included zonal terms of a spherical harmonic gravity model, with various levels of fidelity, and the point mass gravitational attraction of the Earth assumed to follow a circular orbit in the Moon’s equatorial plane. Analytical expressions for the long-term evolution of orbital characteristics are averaged over the mean anomaly and longitude of the ascending node. Later, Yarndley et al. (2020) updated the analytical derivation, predicting multiple frozen lunar orbits with distinct stability exist at some combinations of inclination and altitude in a higher-order lunar gravity field without third-body perturbations.

**Table 2:** Summary of groups of geometrically similar long-lifetime orbits

Group	$i_0$	Subfigures	Perilunes in *pq*-plane	Perilune groundtracks
1	$[5^{\circ },25^{\circ }]$, $[155^{\circ },175^{\circ }]$	7a, g, h; 8a, b; 16c, h, i; 17a, b	q>0, large oscillation in *e* with mean ω near $90^{\circ }$	Limited variation in latitude over northern hemisphere, with knots forming at highest inclinations over $+\hat{X}$ (prograde) or $-\hat{X}$ (retrograde) hemispheres
2	$\{20^{\circ },25^{\circ }\}$, $\{155^{\circ }\}$	7i, 8c; 16g	Encircles e=0 with mean e>0	Encircles both hemispheres
3	$[30^{\circ },45^{\circ }]$, $[135^{\circ },150^{\circ }]$	8g, h, i; 9a; 15h, i; 16a, b	q<0 with mean ω near $-90^{\circ }$, oscillations in *e* and ω increase with decreasing inclination	Limited variation in latitude over southern hemisphere
4	$[50^{\circ },55^{\circ }]$, $[125^{\circ },130^{\circ }]$	9b, c, h; 14i; 15a	Mean q>0, mean ω near $90^{\circ }$	Encircles north pole with knot forming in $+\hat{X}$ (prograde) or $-\hat{X}$ (retrograde) hemisphere
5	$[50^{\circ }]$, $[130^{\circ }]$	9g; 15b, c	Mean q<0, mean ω near $-90^{\circ }$	Encircles south pole, with knot forming in $+\hat{X}$ (prograde) or $-\hat{X}$ (retrograde) hemispheres
6	$[130^{\circ }]$	15g	Encircles e=0 with mean e≠0	Encircles both hemispheres
7	$[60^{\circ },65^{\circ }]$, $[115^{\circ },120^{\circ }]$	9i; 10a; 14g and h	q<0, small variations in *e* and ω, mean ω varies in the range $[240^{\circ },330^{\circ }]$	Limited variation in latitude over southern hemisphere
8	$\{70^{\circ },75^{\circ }, 85^{\circ }\}$, $[95^{\circ },110^{\circ }]$	10g, h, i; 11a, c; 12a, i; 13g, h, i; 14a, b, c	q<0, substantial variations in ω around mean of ω near $-90^{\circ }$	Encircles south pole with knot forming in $+\hat{X}$ (prograde) or $-\hat{X}$ (retrograde) hemisphere and growing in size as *i* approaches $90^{\circ }$
9	$[70^{\circ },85^{\circ }]$, $[95^{\circ },100^{\circ }]$	10b; 11b, g, i; 13a, c	q>0, substantial variations in ω around mean of ω near $90^{\circ }$	Encircles north pole with knot forming in $+\hat{X}$ (prograde) or $-\hat{X}$ (retrograde) hemisphere and varies in size as *i* evolves
10	$\{70^{\circ },80^{\circ },85^{\circ }\}$ and $95^{\circ }$	10c; 11h; 12b; 13b	Encircles e=0	Encircles both hemispheres
11	$90^{\circ }$	12c, g, h	q<0, mean ω near $-90^{\circ }$ with small variations in *e* and ω	Centered over southern pole, shapes possess principal axes offset by $90^{\circ }$ across distinct sets
12	$\{5^{\circ }\}$, $\{175^{\circ }\}$	7b, c; 17c, g	Trace out resonant paths with mean ω near $90^{\circ }$	Limited variation in latitude near zero, with knots forming around $-\hat{X}$ axis for prograde orbits

Figures in Lara (2011) and Yarndley et al. (2020) present the mean value of *q* as a function of inclination for frozen orbits where the mean argument of perilune is $\pm 90^{\circ }$. To approximately compare the results presented in those papers, Fig. 19b displays $q_{center}$, representing the mean value of *q* along each perilune sequence as a function of inclination, colored by the initial semi-major axis. For clarity and further analysis, Fig. 19c displays this information at $a_0=1,788$ km and $a_0=1,938$ km. Note, however, that when calculating $q_{center}$ in this paper, the mean argument of perilune is not necessarily exactly equal to $\pm 90^{\circ }$. Furthermore, the $q_{center}$ curves in Fig. 4 of Yarndley et al. (2020) hold the altitude fixed, as opposed to the semi-major axis held constant in Fig. 19c of this paper and Figs. 1–3 of Lara (2011).

The shapes of the $q_{center}$ curves formed by the long-lifetime orbits presented in this paper are generally consistent with the analytically derived results by Lara (2011) for frozen orbits, with some minor differences. For example, at $a_0=1,938$ km, the discretely sampled values of $q_{center}$ for prograde inclinations in Fig. 19c form a similar shape to the dashed curve in the top subplot of Fig. 1 in Lara (2011). However, at $a_0=1,788$ km, long-lifetime orbits are only recovered for inclinations in the range $i_0=[50^{\circ }, 130^{\circ }]$ and not lower inclinations as predicted in the bottom subplot of Fig. 2 in Lara (2011). Determining whether their absence is due to nonexistence, the sparsity of the generated dataset, their stability, the difference in dynamical models, or the clustering process, is a useful avenue of future work.

This paper presented multiple long-lifetime orbits with the same combination of $i_0$ and $a_0$ due to producing different groundtracks at distinct values of $\Omega _0$. However, in Lara (2011), only one mean value of *q* is reported at each mean inclination; this result was generated by computing the combination of *e* and *i* that satisfy the averaged frozen orbit condition as a function of *a*. For instance, at $a_0=1,788$ km and $i_0=70^{\circ }$, the bottom of Fig. 2 in Lara (2011) identifies one frozen lunar orbit with a mean *q* that is small and negative. In this paper, three long-lifetime orbits with geometrically distinct perilune groundtracks are recovered at distinct, small $q_{center}$ values with distinct values of $\Omega _0$, i.e., initial orientation of the orbit plane in the Moon-fixed frame. Generating a dataset of low lunar trajectories with a finer resolution in $\Omega _0$ as well as longer durations would support determining whether the multiple long-lifetime orbits recovered in this paper (1) are likely to exist in a continuous family or discrete sets and (2) retain a low drift over longer durations. Note that three distinct mean *q* curves are computed for perilunes with an altitude of 50 km near $i_0=70^{\circ }$ in Fig. 4 of Yarndley et al. (2020), producing two unstable and one stable frozen orbits. However, the mean values of *q* for the unstable orbits possess larger magnitudes than those recovered in this paper. Thus, the two additional long-lifetime orbits in this paper are not expected to exist near the two unstable frozen orbits in Yarndley et al. (2020).

#### Comparison to analytically predicted mean eccentricity and mean argument of perilune

Prior analytical approaches in the literature have predicted that perilune sequences of lunar frozen orbits tend to possess mean values of *e* and ω that fall into one of the three categories. These three cases include: (1) a mean argument of perilune equal to $\pm 90^{\circ }$ (Park and Junkins 1994; Lara 2011), (2) a mean eccentricity of zero (Nie and Gurfil 2018), or (3) a mean argument of perilune equal to $0^{\circ }$ or $180^{\circ }$ (Nie and Gurfil 2018). Comparing the results in this paper to these predicted cases reveals:

1. Most of the long-lifetime orbits displayed in Figs. 7, 8, 9, 10, 11, 12, 13, 14, 15, 16 and 17 possess average arguments of perilune close to $\omega =\pm 90^{\circ }$ or encircle e=0, demonstrating approximate consistency with the first two cases.
2. Long-lifetime trajectories with a mean argument of perilune equal to $0^{\circ }$ or $180^{\circ }$ and low drift do not appear in Figs.  7, 8, 9, 10, 11, 12, 13, 14, 15, 16 and 17. In Fig. 6 of Nie and Gurfil (2018), frozen orbits with these mean values of ω exist at a limited range of average inclinations near $63.43^{\circ }$ and $116.57^{\circ }$ at similar semi-major axes. However, in this paper, the sparse sampling of the inclination at $10^{\circ }$ increments may potentially cause these low drift long-lifetime orbits to not be recovered, even if they exist in a high-fidelity ephemeris model. Further examining this case with a finer sampling of initial conditions is an interesting avenue of future work.
3. Some of the long-lifetime orbits selected at $i_0=65^{\circ }$ and $i_0=115^{\circ }$ in Figs. 10a and 14g are not consistent with any of the three cases. Specifically, trajectories with a low drift in the *pq*-plane over 180 days also possess average arguments of perilune near $240^{\circ }$, $300^{\circ }$, and $330^{\circ }$ when $a_0=1,988$ km or $a_0=2,038$ km.

**Fig. 20:**
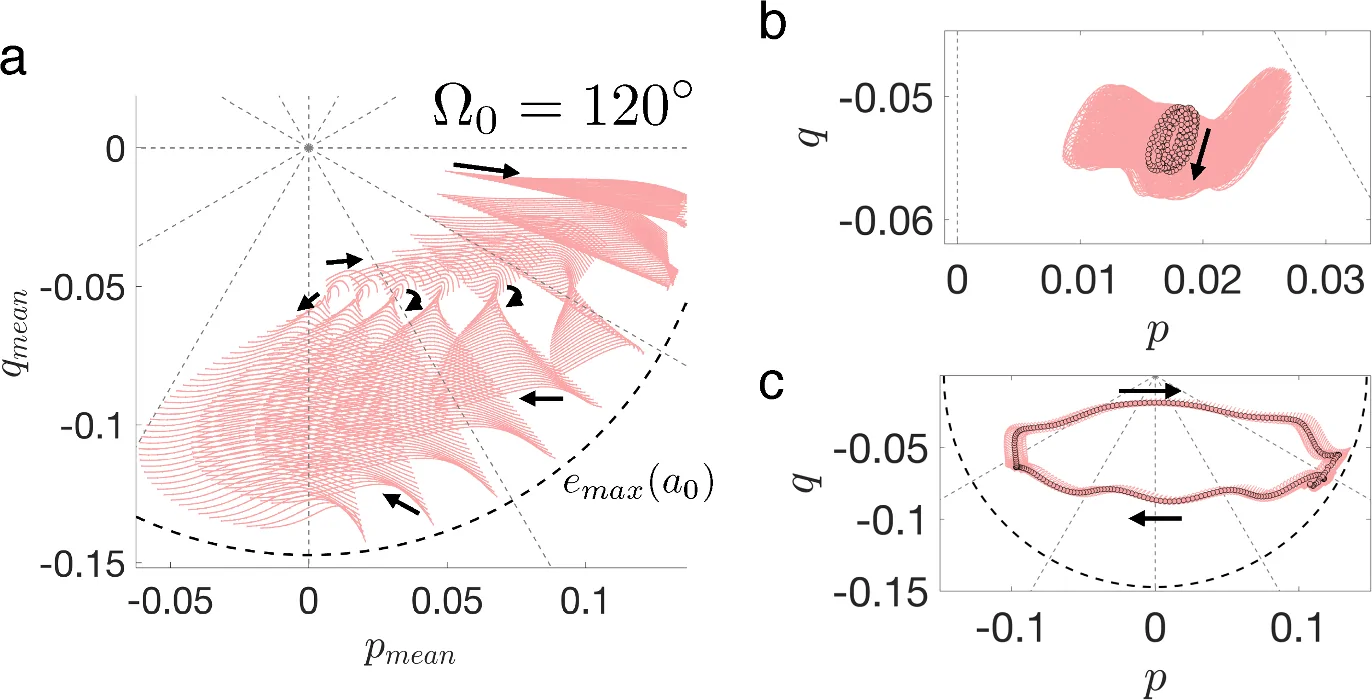
Perilune sequences at $a_0=2,038$ km, $i_0=115^{\circ }$, $\Omega _0=120^{\circ }$: **a** evolution of $p_{mean}$ and $q_{mean}$ for $e_0\in [0.05, 0.15]$ and $\omega _0\in [240^{\circ }, 350^{\circ }]$ over two years, as well as instantaneous and mean values of *p* and *q* over 19.73 years for **b** $e_0=0.06$ and $\omega _0=290^{\circ }$ and **c** $e_0=0.14$ and $\omega _0=330^{\circ }$

To investigate the inconsistency between the presented results and analytically predicted frozen orbits at $i_0=65^{\circ }$ and $i_0=115^{\circ }$, consider a denser set of trajectories generated for up to two years with $a_0=2,038$ km, $i_0=115^{\circ }$, $\Omega _0=120^{\circ }$, $e_0\in [0.05, 0.15]$ with a step-size of $2\times 10^{-3}$, and $\omega _0\in [240^{\circ }, 350^{\circ }]$ with a step-size of $10^{\circ }$. The values of $p_{mean}$ and $q_{mean}$ along their perilune sequences are plotted in Fig. 20a. Due to the size and density of this dataset, these discrete quantities are connected using curves for visual clarity. The mean values of *p* and *q* along the generated perilune sequences appear to be governed by at least two dominant frequencies with loops forming near $q_{mean}=-0.054$. Some sequences in this region, with mean arguments of perilune between $270^{\circ }$ and $300^{\circ }$, oscillate with a tightly bounded path in the *pq*-plane. Interesting avenues of future work include 1) identifying whether additional frozen orbits may exist at other locations in the *pq*-plane for $i_0=65^{\circ }$ or $i_0=115^{\circ }$, and 2) examining these more complex oscillations in $p_{mean}$ and $q_{mean}$.

Some of the 180-day long-lifetime orbits identified at $i_0=65^{\circ }$ and $i_0=115^{\circ }$ produce perilune sequences that remain tightly bounded over longer durations with a mean argument of perilune that differs substantially from $-90^{\circ }$. An example perilune sequence with $e_0=0.06$ and $\omega _0=290^{\circ }$ is generated for 19.73 years and plotted in Fig. 20b: the instantaneous values of *p* and *q* are plotted using thin curves whereas the values of $p_{mean}$ and $q_{mean}$ along each revolution are plotted using large circles outlined in black. This trajectory resembles a frozen orbit but possesses a mean argument of perilune that is $18^{\circ }$ away from the $\omega =-90^{\circ }$ analytical prediction. Further investigation and explanation of this discrepancy is an interesting avenue of future work.

Other 180-day long-lifetime orbits identified at $i_0=65^{\circ }$ and $i_0=115^{\circ }$ produce perilune sequences that drift over longer durations due to longer period oscillations. Consider a perilune sequence generated for 19.73 years with $e_0=0.14$ and $\omega _0=330^{\circ }$, i.e., the initial conditions of one of the long-lifetime orbits plotted in Fig. 14g. In Fig. 20c, the first few revolutions of the associated perilune sequence form a small loop in $p_{mean}$ and $q_{mean}$ and, therefore, exhibit limited drift over 180 days. However, over longer durations, larger oscillations occur. Although this trajectory is not expected to exist near a frozen orbit, the perilune sequence exhibits a large libration in the *pq*-plane over 19.73 years, potentially governed by at least two dominant frequencies.

#### Comparison to existing numerical results

Resonant perilune sequences in the *pq*-plane tend to be excluded from analytical predictions in the existing literature due to mathematical requirements in the derivations. However, some numerical approaches do not suffer from similar restrictions. For instance, Russell and Lara (2007) used differential corrections to recover low lunar trajectories with high-order resonances in a lower-fidelity dynamical model to produce repeat groundtrack orbits. In contrast, the numerical exploration in this paper recovered perilune sequences exhibiting low-order resonances in the *pq*-plane at low inclinations. Examples appear in Figs. 7b–c and 17c and g.

Some of the long-lifetime orbits presented in this paper have also been generated numerically by previous researchers. For example, the long-lifetime orbits in Fig. 12i at $i_0=95^{\circ }$ possess a similar shape to the 328-cycle repeat groundtrack orbit with a mean inclination of $92.98^{\circ }$ generated by Russell and Lara (2007); the difference in the size of the paths in the *pq*-plane is expected to be due to the differences in inclination and dynamical model. The values of $T_{osc}\in [27.43,27.54]$ days at $i_0=95^{\circ }$ are also similar to the reported period of 27.35 days in Table 2 of Russell and Lara (2007). The perilune groundtracks in Fig. 12g at $i_0=80^{\circ }$ also resemble those at a mean inclination of $83.2^{\circ }$ in Fig. 13 of Russell and Lara (2007). Additionally, Figs. 8g, 9i, and 12c display long-lifetime orbits that possess similar inclinations, semi-major axes, and paths in the *pq*-plane to the frozen orbits in Fig. 14 of Folta and Quinn (2006). Furthermore, the prograde orbits in Fig. 18a–c resemble the near-equatorial orbits in Figs. 15 and 17 of Plice et al. (2017).

### Future work

These results supply a foundation for future work. For instance, a dataset could be generated across more finely sampled initial conditions, additional epochs, models of distinct fidelity, or longer durations. A broader set of clusters could then support identifying the persistence of geometrically distinct long-lifetime orbits across these parameters. This dataset could also be generated adaptively to support efficiently balancing global and local coverage of distinct regions of the phase space, while minimizing the dataset size. Using these more generalizable datasets, a finer comparison to existing analytical theories could be performed. Furthermore, this procedure may be useful for exploratory analysis of long-lifetime orbits in other dynamical environments.

## Conclusions

This paper presents a new clustering approach to summarize a large set of low lunar trajectories by their evolution of perilune. First, a wide variety of trajectories are numerically propagated for 180 days in a high-fidelity lunar gravity model with the point mass gravity of the Earth and Sun, given a fixed initial epoch. Then, the trajectories are input to a distributed trajectory clustering algorithm to create groups of trajectories with perilunes that evolve with a similar geometry, regardless of their initial phasing. The result of this process is a global cluster summary of the original dataset, that reduces the amount of information for exploratory analysis.

From this summary, sets of long-lifetime orbits are extracted at each initial semi-major axis from selected global clusters as trajectories with (1) a low drift over 180 days and (2) a similar perilune groundtrack. These long-lifetime orbits are then compared with existing analytical and numerical results on frozen orbits that persist over longer durations. Most long-lifetime orbit sets possess a similar average eccentricity and inclination as a function of semi-major axis to analytical predictions of frozen orbits. However, the average eccentricity of long-lifetime orbits in the same global cluster can vary as a function of initial longitude of the ascending node in the Moon-fixed frame, sometimes producing changes in the perilune groundtrack. Furthermore, most long-lifetime orbits possess an average argument of perilune of $\pm 90^{\circ }$ or average eccentricity of zero, consistent with these frozen orbit predictions. However, at initial inclinations of $65^{\circ }$ and $115^{\circ }$ some long-lifetime orbits possess a mean argument of perilune that differs substantially from $-90^{\circ }$ by up to $30^{\circ }-60^{\circ }$. Some trajectories at these inclinations are demonstrated to maintain a low drift over much longer durations, indicating the existence of nearby frozen orbits, whereas others drift substantially after 180 days while governed by longer period oscillations. In addition, long-lifetime orbits that exhibit a resonance in eccentricity and argument of perilune are recovered at low inclinations.

## Supplementary Information

Below is the link to the electronic supplementary material.

Supplementary file 1 (pdf 93 KB)

Supplementary file 2 (txt 28 KB)

## Data Availability

The initial conditions and global clusters of the long-lifetime orbits presented in this paper are provided in a supplementary text file (Online Resource 1).

## Declarations

### Code availability

No additional codes are provided.
