# Supplementary material for: Numerical exploration of low lunar, long-lifetime orbits
Source: Celest Mech Dyn Astron. 2026 Oct 4;138(5):61. doi: 10.1007/s10569-026-10334-x (PMC13635055; doi:10.1007/s10569-026-10334-x)
Supplement: Supplementary file 1 [file 10569_2026_10334_MOESM1_ESM.pdf]

The following table summarizes the parameters governing the clustering process that is described in the following manuscript: Bosanac, N., Miceli, G.E., ‘Numerical Exploration of Low Lunar, Long-Lifetime Orbits’, Celestial Mechanics and Dynamical Astronomy, 2026.

Table 1 Table of parameters governing the clustering process

**Governing parameter**	**Influence or rationale for selection**	**Value**
Δt, time interval for sampling perilunes from a sequence	Smaller value increases the number of perilunes used to represent the trajectory, along with computational time and storage	0.5 days
Duration of subsequence used to estimate drift and period of oscillation	Must be less than minimum possible period of oscillation	14 days
N_{thresh}, number of nonconsecutive sampled perilunes that must lie within dense, connected neighbors for two trajectories to be grouped	Larger value accommodates larger differences in duration or brief shape differences, but can result in geometrically distinct trajectories being grouped incorrectly	$N_{max} - 27$, where N_{max} is maximum number of perilunes
m_{pts}, number of members that must lie in a neighborhood for sufficient density	Smaller values prioritize discovering local variations across a sparse dataset, at the expense of potentially smaller clusters	4
ϵ, the radius of the m_{pts}-neighborhood used to identify a core point	Select heuristically, where possible, and large enough to encompass m_{pts} neighbors in a sparsely sampled dataset	$2\sin(10°)$
k_1 and k_2, number of candidate local clusters to consider when merging across datasets and initial phasing, respectively	Larger values consider more clusters for potential merging, but increase the computational time	2 and 4, respectively

Supplementary material for: Bosanac, N., Miceli, G.E., ‘Numerical Exploration of Low Lunar, Long-Lifetime Orbits’, Celestial Mechanics and Dynamical Astronomy, 2026.
